# Optimising the Efficacy of Equine Welfare Communications: Do Equine Stakeholders Differ in Their Information-Seeking Behaviour and Communication Preferences?

**DOI:** 10.3390/ani10010021

**Published:** 2019-12-20

**Authors:** Persephone Pickering, Jo Hockenhull

**Affiliations:** Animal Welfare and Behaviour Group, Bristol Veterinary School, University of Bristol, Langford, Bristol BS40 5DU, UK

**Keywords:** dissemination, horse welfare, knowledge transfer, veterinarian, yard owner

## Abstract

**Simple Summary:**

To ensure that up to date equine welfare advice is communicated as effectively as possible, it is important to understand where equine stakeholders seek information and their preferences for the way that information is communicated. In this study we surveyed horse owners, equine veterinarians, and livery yard owners. We found that although the three groups differed in the information sources they used most frequently, there was a tendency to consult other people rather than organisations, or digital and printed resources. There was a preference for information to be communicated in a neutral or positive way. Horse owners in particular would like practical information on the process of implementing equine welfare improvements included alongside the information. Equine veterinarians are important sources of information for all three stakeholder groups.

**Abstract:**

Information on the management of animals within domestic environments is freely available to animal owners and caregivers either online, or in paper form by request. However, awareness is growing within the animal welfare sector that simply providing written guidelines or educational material is not enough to affect a positive change in owners in relation to animal welfare. In the quest to improve equine welfare, understanding the way that owners and other stakeholders seek information and their communication preferences is key to effective dissemination of up to date equine welfare information and research findings. Three UK equine stakeholder groups—horse owners, livery yard owners, and equine veterinarians—were surveyed online to find out where they sought equine information. Their awareness of equine welfare Codes of Practice, how they respond when they are asked to give advice to horse owners and their communication preferences were included within the survey. All three stakeholder groups tended to seek information from people rather than from organisations, or digital and printed resources. Veterinarians were the most used information source across all three stakeholder groups This highlighted the importance of ensuring that equine veterinarians have access to up to date, evidence-based equine welfare information. While the majority of participants were aware of the equine welfare Code of Practice, fewer had actually read it, this was true particularly amongst horse owners. The primary reasons for this were the features of the Code as well as the issuing organisation. The stakeholders expressed a preference for information to be communicated in a neutral or positive way rather than focusing on negative aspects. Our findings suggest that industry professionals, particularly veterinarians, have an important role to play in knowledge transfer and the dissemination of research findings to horse owners. The efficacy of equine welfare communication could be improved if the information delivery preferences of equine stakeholders are were taken into consideration.

## 1. Introduction

There is a growing body of evidence-based knowledge on the impact of different husbandry and training practices on equine welfare. The challenge lies in the process of turning this knowledge base into tangible improvements in our interactions with, and management of, equines in a domestic setting. The process of changing human behaviour is a science in itself [1,2] and is increasingly being drawn upon by those seeking to improve animal welfare [3,4,5,6]. Before the process of changing human behaviour can begin, stakeholders need access to information to raise their awareness that a problem exists and to give them the knowledge of what the potential solutions may be [1]. To achieve this, the body of evidence-based knowledge needs to move from the realm of animal welfare scientists and researchers into the public domain.

Information on how to manage animals within domestic environments is typically available to owners and caregivers in the form of both guidelines or as recommendation documents freely available online or in paper format by request. However, the efficacy of this form of knowledge transfer is largely unknown. These guidelines and recommendation documents generally outline minimum standards that should be met across different aspects of the animal’s management to prevent poor welfare. The equine sector is no different, in the UK the Code of Practice for the Welfare of Horses, Ponies, Donkeys and their Hybrids (hereafter the DCP) is published by the Department for Environment, Food and Rural Affairs (DEFRA) [7]. While failure to meet the requirements of the DCP is not an offence in itself, if an owner is taken to court on animal welfare charges, their compliance with the DCP will be used to determine whether an offence has been committed. A lack of awareness of the DCP therefore could have serious implications.

Mismanagement through owner ignorance is perceived to be a greater cause of poor welfare within equines than intentional harm [5]. However, utilisation of equine welfare information resources is dependent on several factors: stakeholders being aware that they exist, their motivation to seek them out and then to follow the recommendations given [8]. Due to the devolved parliaments within the UK, multiple Codes of Practice exist alongside that published by DEFRA; the governments of Scotland, Wales, and Northern Ireland have all separately published their own equine welfare Codes [9,10,11]. In addition to these Codes of Practice, the UK also has Equine Industry Welfare Guidelines published by the National Equine Welfare Council [12], alongside guidelines and advice published by animal welfare and equine welfare focused charities. Yet, despite the number of guideline and recommendation documents available, it is not understood whether this is an effective way of disseminating knowledge to equine owners and caregivers.

Despite the plethora of equine welfare information available online and the best intentions of owners, the welfare of many horses is still compromised. This occurs often due to a lack of knowledge or practical application of recent equine welfare research findings [13]. It is unclear whether horse owners and other equine stakeholders are aware the equine welfare Codes of Practice exist, and perhaps more saliently, whether or not they make use of them.

Understanding where equine stakeholders are seeking information, and their preferences for different communication styles, is vital to ensuring government organisations and welfare charities concentrate their resources effectively for equine welfare improvement. Furthermore, this understanding is necessary for research findings to reach the very population that can benefit from them. Research on human behaviour change has shown that simply making information accessible does not mean that people will access it, engage with it, or that it will result in a change in practices [2]. This is especially true when it comes to trying to promote changes that will improve animal welfare, as the beneficiary of the change is a third party [3]. Discovering better ways of communicating up-to-date equine welfare information to horse owners would help ensure better welfare for horses in the future.

The aim of this study was to investigate the information-seeking behaviour and communication preferences of three key equine stakeholder groups in the UK (horse owners, veterinarians, and livery yard owners) with the ultimate goal to improve the efficacy of communicating equine welfare information. A large proportion of leisure horses in the UK are kept at livery yards where owners pay for their horses housing or their day to day care depending on the type of yard. Previous research suggested that livery yard owners (hereafter yard owners) maybe a valued source of information for people who keep their horse(s) in these establishments [14]. Veterinarians are another commonly used information source. Understanding where these information gatekeepers source their information is also critical for maximising the impact of equine welfare research dissemination.

## 2. Materials and Methods

The data presented in this study were derived from a larger data set generated by a cross-sectional survey conducted between 7 February 2017 and 12 March 2017. The survey specifically targeted three distinct UK equine stakeholder groups: horse owners (which also included people who loaned or regularly cared for a horse owned by someone else), equine veterinarians and yard owners. The survey was conducted online through the Bristol Online Survey software (BOS) and was approved by the University of Bristol Faculty of Health Sciences Ethical Committee for research with human subjects (sub-committee for student projects).

### 2.1. The Survey

The survey incorporated closed and open-ended questions, which resulted in both quantitative and qualitative data. This was intended to generate a greater depth of detail and understanding than would be the case using closed questions alone.

**Figure 1:**
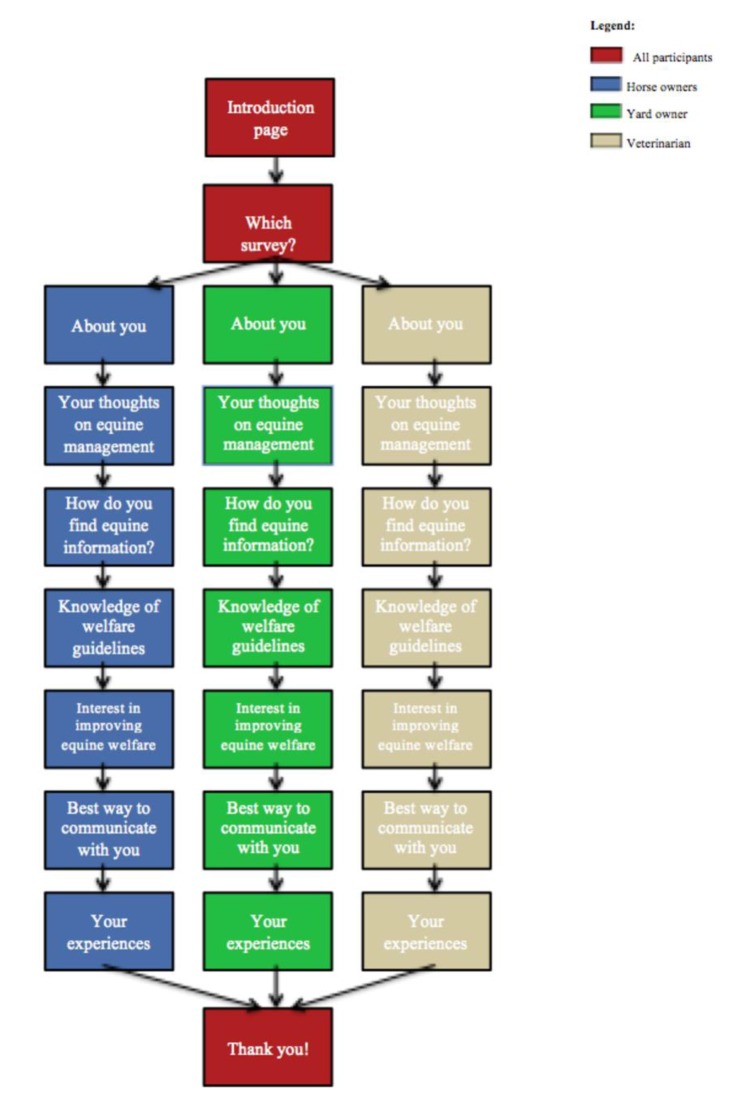
Flow chart of the structure of all three versions of the online survey completed by the three stakeholder groups; horse owners, yard owners, and equine veterinarians.

The survey link took participants to an information page and once they accepted the terms and conditions, and thereby gave their informed consent to participate, they were directed to the next page where they chose the survey for their stakeholder group: the one for horse owners, yard owners or equine veterinarians. Each survey contained similar questions, although the wording differed for each stakeholder group solely to reflect their different roles within the equine sector. For example, while horse owners were asked how long they had owned/looked after horses, the equine veterinarians were additionally asked how many years they had been a vet, and similarly livery yard owners were asked how long they had owned or managed livery yards. The structure of the full survey for each stakeholder group is depicted in Figure 1 and the full versions of all three surveys can be found in the supplementary materials (Survey S1).

The surveys were piloted by representatives from all three stakeholder groups leading to changes in phraseology and question format, e.g., incorporating individual related questions into a matrix format.

The full surveys contained 37 questions, covering respondent demographics, and their views and experiences of trying to improve equine welfare and source information. The data presented here relate to respondent age, information-seeking behaviour, knowledge of equine welfare guidelines, and communication preferences and were collected in five sets of questions that were identical in the three versions of the survey completed by the stakeholder groups.

### 2.2. Respondent Age

At the start of the survey, respondents were select which age category they belonged to from the options 16–25 years, 26–45 years, 46–65 years, and 65+ years.

### 2.3. Information-Seeking Behaviour

The set of questions used to explore information-seeking behaviour presented six different scenarios covering health, diet, management, training, behaviour and welfare (see supplementary material for full wording) in a matrix format. For each scenario the respondent was asked to select the top three information resources that that would be most likely to consult, and the one source that they would be least likely to consult. Respondents were given a drop-down list of 17 information sources to choose from: riding instructor, veterinarian, farrier, yard owner, equine charity, equine physiotherapist, animal behaviourist, tack shop worker, horse owner, course I have followed/am following, equine magazine, internet forums, online searches, scientific journals, books, DEFRA Code of Practice or similar guidelines, and other. The 17 information sources presented were the same for each scenario and each stakeholder group. The six scenarios were followed by a closed question asking respondents how frequently on average they searched for information. There were six answer options to choose from; weekly, monthly, several times a year, once a year, hardly ever, and never.

### 2.4. Giving Advice

A matrix question was used to gain data on whether the respondent gave advice to (other) horse owners. The same six subject areas were given as covered in the scenarios in the previous set of questions on information-seeking behaviour. For each subject the respondent was asked to tick whether it was something they had given advice on themselves and/or whether they had been asked about it and had referred the enquirer to another source of information instead of or in addition to giving advice themselves.

### 2.5. Awareness of the Defra Code of Practice (DCP)

Knowledge of the DEFRA Code of Practice for the Welfare of Horses, Ponies, Donkeys and their Hybrids (DCP) was explored in a set of three binary radio questions, with the respondent asked to select ‘yes’ or ‘no’ to whether they knew of the DCP, whether they had read the DCP and whether they had looked at any other guidelines or recommendations. If they selected ‘yes’, there was an open text box where they were asked to specify what these were.

### 2.6. Communication Preferences

The question set on communication preferences presented statements of advice relating to provision of water, diet, and bedding. These three pieces of advice were taken from the DCP. However, in the questionnaire the advice was presented in three ways, one was written negatively (stating the problems caused if the advice is not followed), one written neutrally (simply giving the advice), and one written positively where the benefits of following the advice were given. Respondents were asked to select which statement they would be most likely to follow using a closed (radio button question) and why (open text question) for each of the three subjects. Following on from this, respondents were asked what explanations or evidence they would want to have included if they were presented with details of a new method for managing horses to make them more likely to try or suggest this method. This was formatted as a tick-all-that-apply question, with eight options given: information showing the benefit of this new method, information showing the negative effects of not applying this method, information stating what method you should use with no need for explanations, evidence taken from scientific research papers, examples of specific horses that have suffered from other methods, examples of specific horses that have thrived with the new method, advice on how to implement this method and other. The respondents were then asked why they had chosen these options.

### 2.7. Recruitment of Respondents

At the time the survey was conducted, there was no central database for horses and their owners in the UK [15], making studying this population challenging. This survey was distributed by a number of different strategies; via social media using Facebook through public sharing (with 582 shares), posting in equine groups and private messages to equine veterinarian companies and bloggers. It was also distributed through MailChimp mailing lists for the yard owners (471 British Horse Society (BHS) approved lively yards emails), equine veterinarians (284 Royal College of Veterinary Surgeons (RCVS) and British Equine Veterinary Association (BEVA) emails). This enabled the use of personalised emails and prevented them going into recipients’ spam folders. Emailed requests targeted at yard owners and veterinarians specifically requested that they complete these versions of the survey whether or not they also owned a horse. A follow-up email was sent three weeks later (as Hotchkiss et al.’s [16] study did via post) requesting more participants and thanking those that had taken part. The use of a reminder has been found to increase response proportions [17,18].

## 3. Analysis

Data were exported from the Bristol Online Survey Software (BOS) to an Excel (Microsoft Office 365 ProPlus, Microsoft Corporation, Redmond, WA, USA) spreadsheet where they were cleaned prior to analysis. This involved removing respondents that were not living in the UK as identified from their postcode information that was collected in the survey section requesting demographic information.

Only a subset of the survey data from the three stakeholders is reported here as not all data were relevant for the focus of this analysis. There is also variation in the number of respondents answering each question as, apart from selecting which survey they wanted to take, there was no mandatory answering required by the respondent to progress through the survey. This has resulted in a variation in the N values reported for each question.

Due to the large discrepancy in sample sizes between the three equine stakeholder groups and the categorial nature of the data, the quantitative data were analysed using Pearson χ^2^ tests for independence in IBM SPSS Statistics (version 23) for Mac (SPSS Inc., Chicago, IL, USA).

The qualitative data were analysed manually in NVivo for Mac (Version: 10.2.2), noting common themes that reoccurred in the answers. Then the frequency each theme appeared was recorded and the percentage calculated out of the total number of respondents for that question.

## 4. Results

The survey was completed by 511 horse owners, 64 yard owners, and 35 veterinarians; a total of 610 participants.

### 4.1. Respondent Age

The three stakeholder groups differed significantly in respondent age (χ^2^ = 35.94, df = 6, *p* < 0.001). The horse owners had a median age of 26–45 years old, as did the veterinarians. The median age of the yard owners was 46–55 years old.

### 4.2. Information-Seeking Behaviour

There was a significant difference between stakeholder groups in how frequently they search for information (χ^2^ = 18.96, df = 10, *p* = 0.041). Veterinarians searched for information more regularly (median = weekly), compared to horse owners and yard owners (median = monthly for both).

It is important to note that the respondents could select up to three sources that they are likely to consult for each scenario; many respondents only selected one or two, which has led to variation in the number of responses for each scenario.

**Figure 2:**
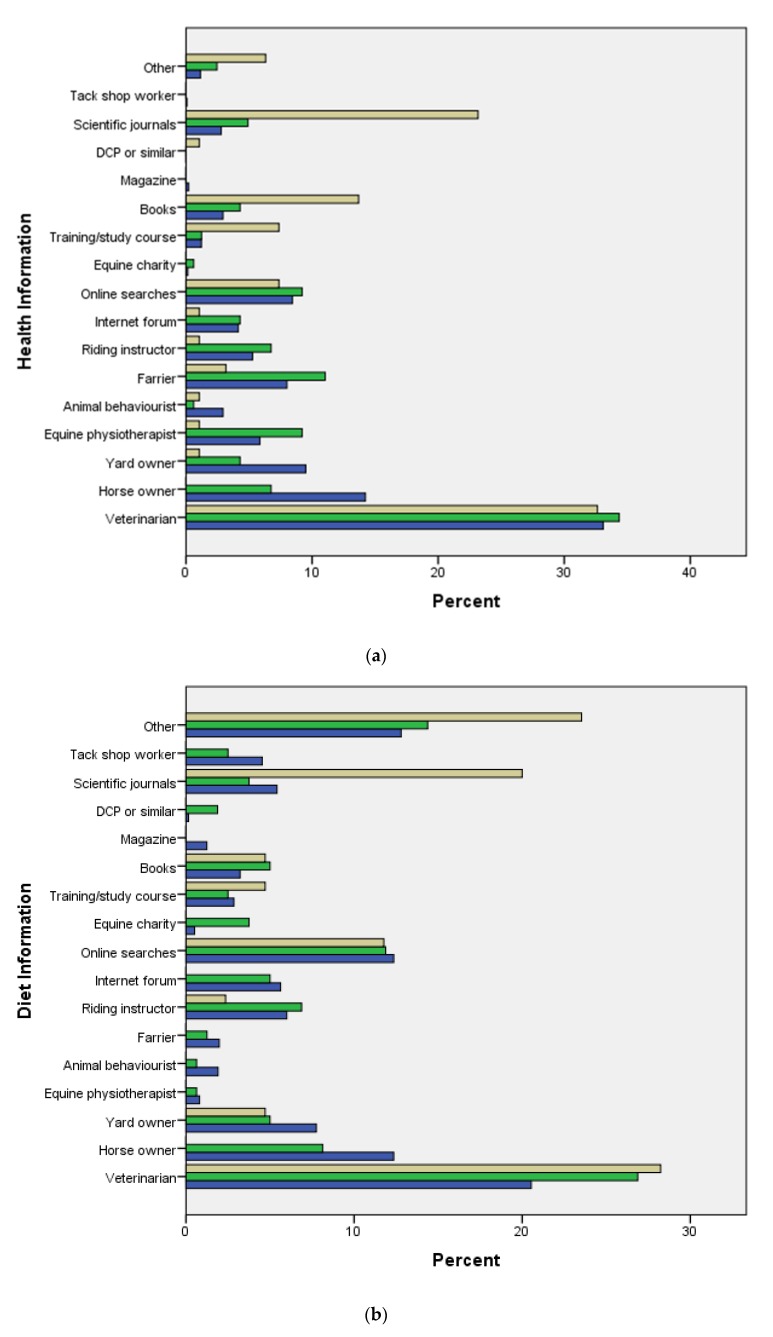

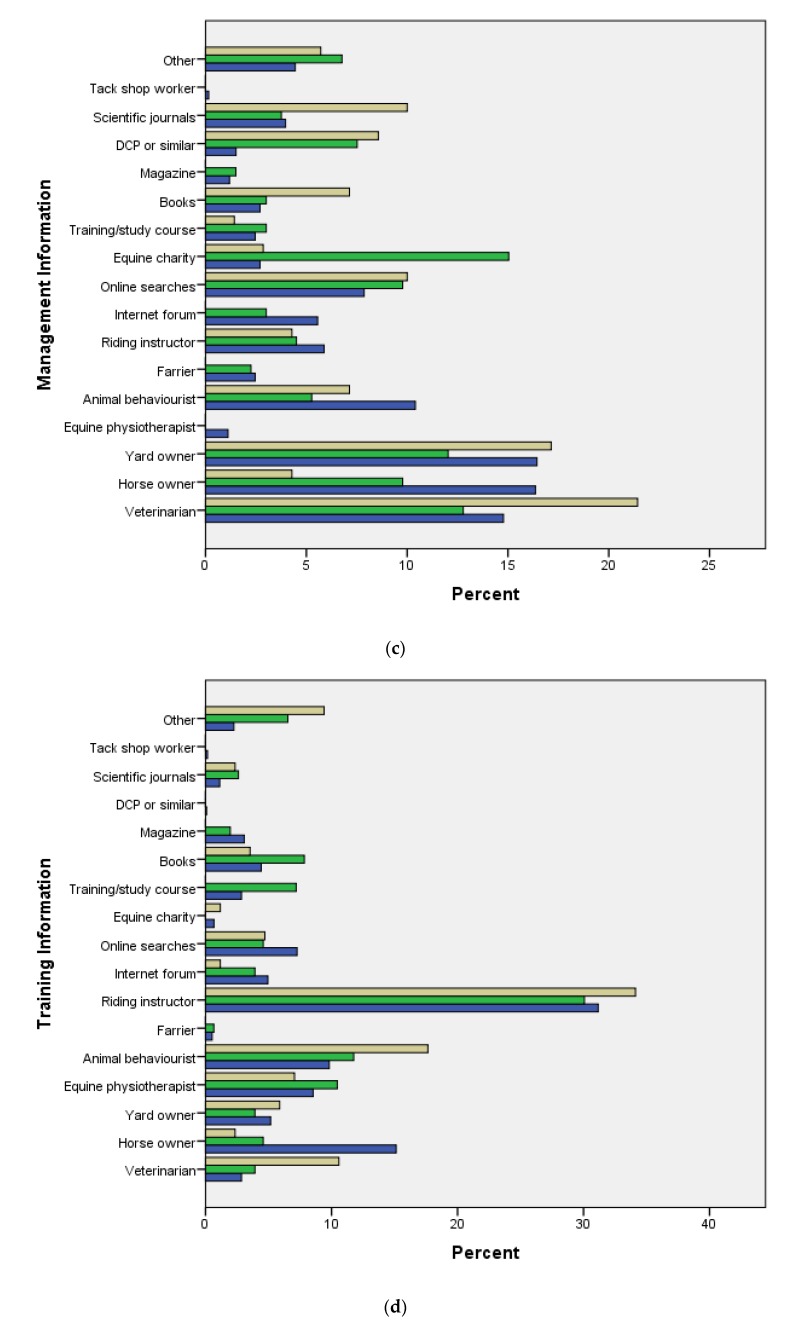

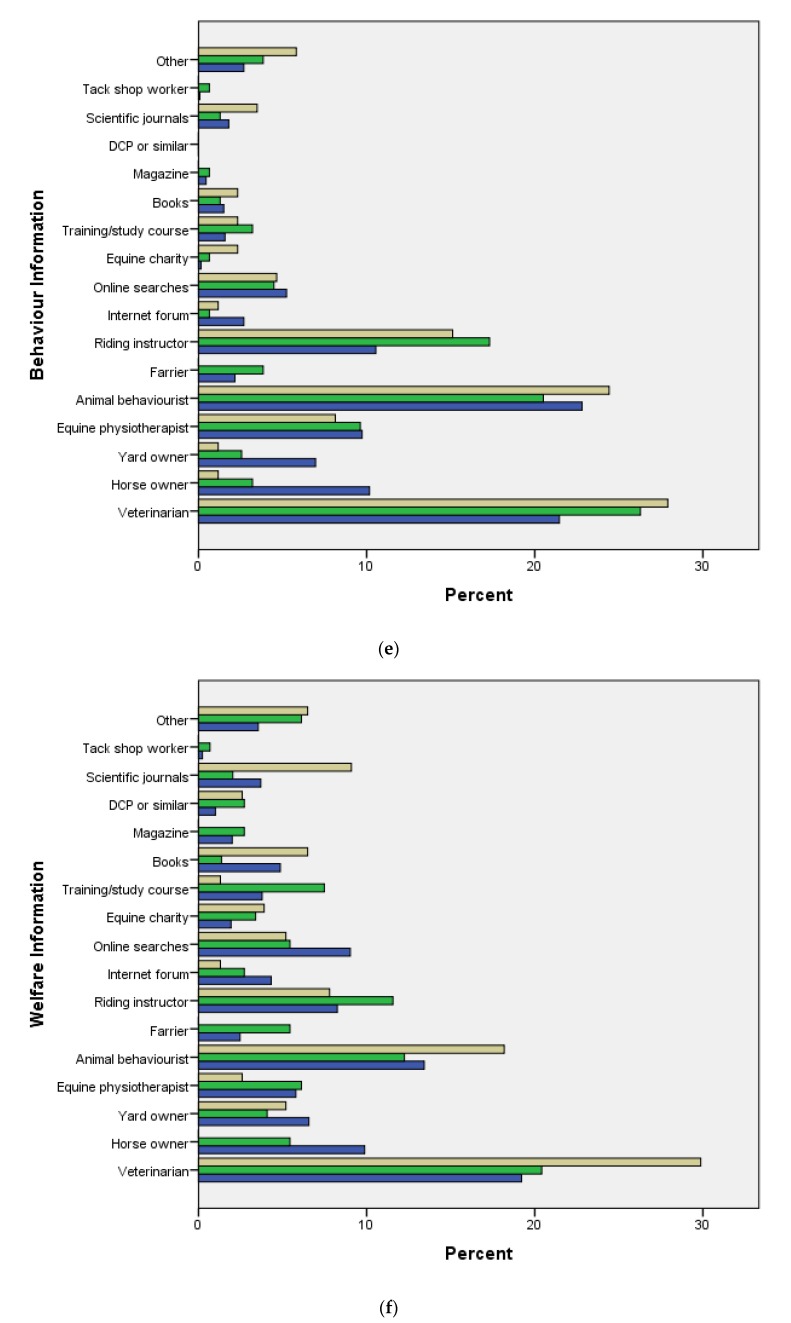
(**a**) Percentage of horse owners (blue), yard owners (green), and veterinarians (beige) selecting each information source as the sources that they are most likely to consult for health information (χ^2^ = 226.09, df = 32, *p* < 0.001). (**b**) Percentage of horse owners (blue), yard owners (green), and veterinarians (beige) selecting each information source as the sources that they are most likely to consult for diet information (χ^2^ = 112.98, df = 32, *p* < 0.001). (**c**) Percentage of horse owners (blue), yard owners (green), and veterinarians (beige) selecting each information source as the sources that they are most likely to consult for management information (χ^2^ = 118.98, df = 32, *p* < 0.001). (**d**) Percentage of horse owners (blue), yard owners (green), and veterinarians (beige) selecting each information source as the sources that they are most likely to consult for training information (χ^2^ = 88.84, df = 32, *p* < 0.001). (**e**) Percentage of horse owners (blue), yard owners (green), and veterinarians (beige) selecting each information source as the sources that they are most likely to consult for behaviour information (χ^2^ = 63.01, df = 32, *p* < 0.001). (**f**) Percentage of horse owners (blue), yard owners (green), and veterinarians (beige) selecting each information source as the sources that they are most likely to consult for welfare information (χ^2^ = 62.70, df = 32, *p* = 0.001).

There were significant differences between the information sources that each stakeholder group was most likely to consult for each of the six scenarios presented (Figure 2a–f).

Across the six scenarios, veterinarians were the information source most likely to be consulted by all three equine stakeholder groups, chosen by 18.7% (1581/8456) of horse owners, 20.8% (200/961) of yard owners and 25.6% of veterinarians (132/516)). The second most frequently chosen source differs for each type of respondent: 13.1% (1104/8456) of horse owners consulted other horse owners, yard owners chose riding instructors as their second most consulted information source (12.8% (123/961)) and 11.4% (59/516) of veterinarians consulted animal behaviourists while the same number consulted scientific journals

Survey respondents were also asked to pick the information source that they would be least likely to consult if faced with the same six different scenarios. The three stakeholder groups did not significantly differ in the sources that they were least likely to consult for each subject area. All three stakeholder groups chose tack shop workers as the information source that they were least likely to consult across all six scenarios; horse owners (18.7% (485/2599)) veterinarians (32.9% (51/155)), although for yard owners this was jointly tied with internet forums (both chosen by 15.5% (48/309)).

### 4.3. Giving Advice

**Figure 3:**
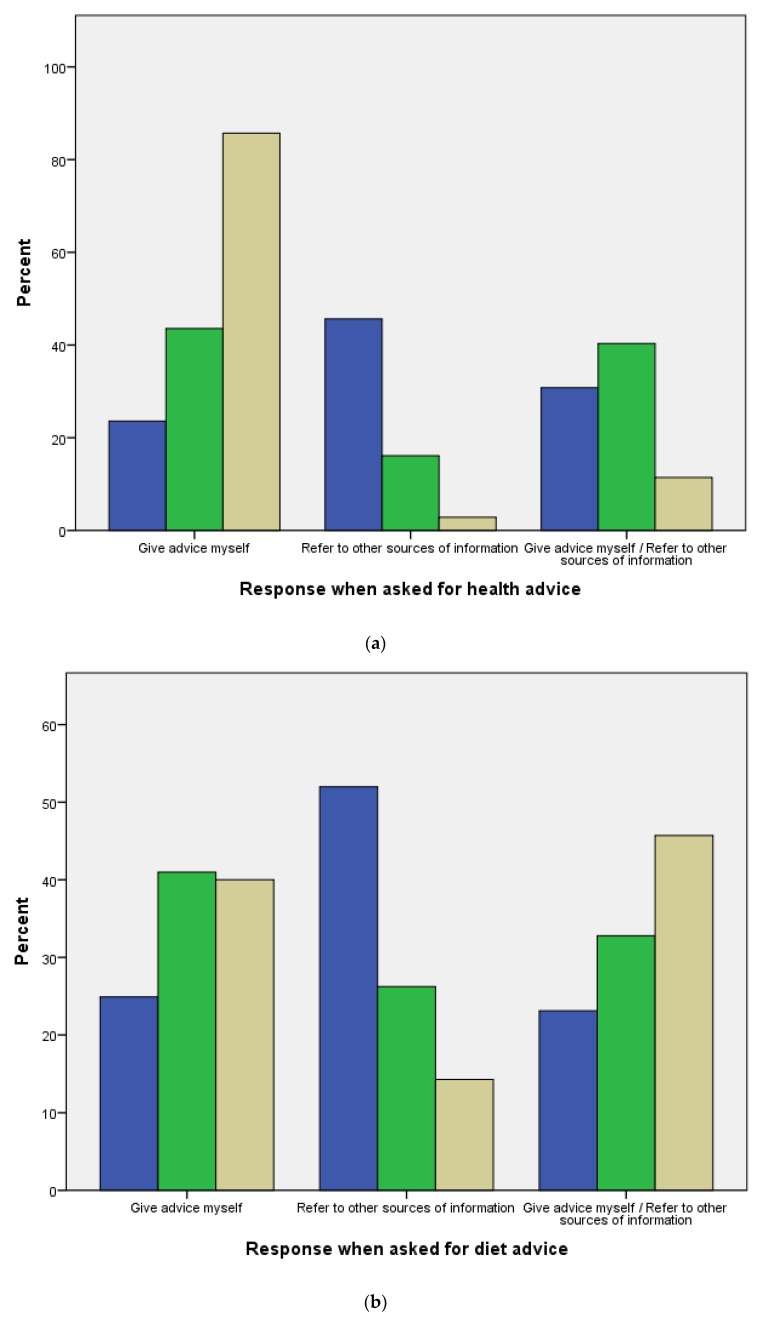

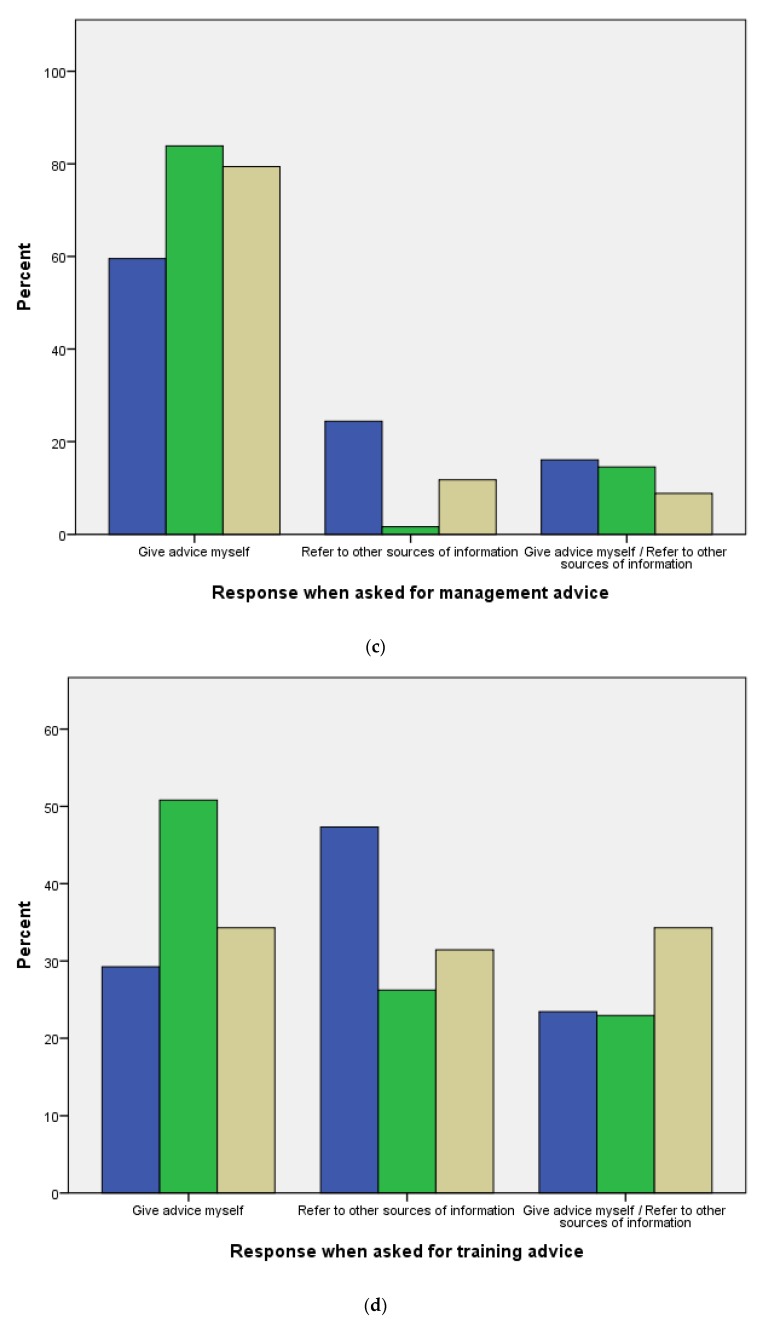

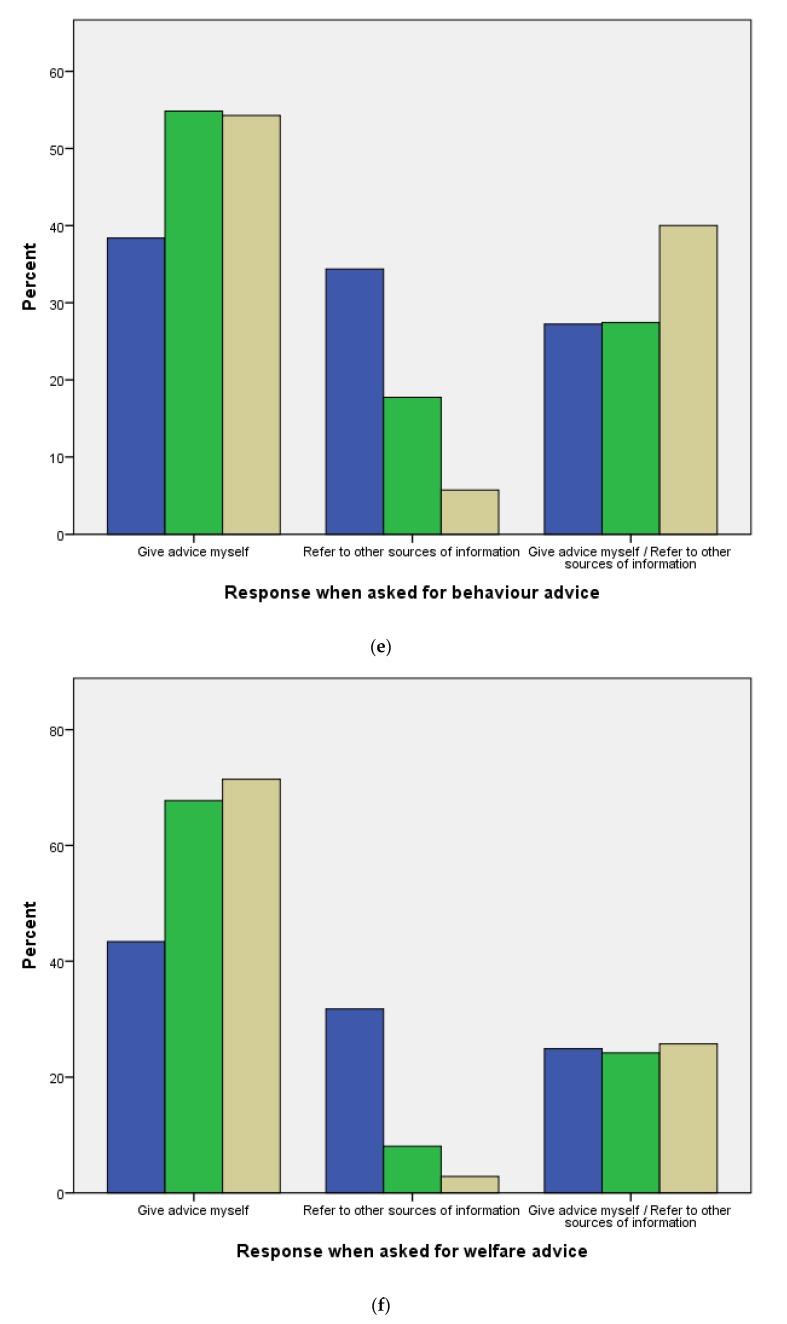
(**a**) Percentage of horse owners (blue), yard owners (green), and veterinarians (beige) giving advice, referring people to other information sources or both when asked for health information (χ^2^ = 77.57, df = 4, *p* < 0.001). (**b**) Percentage of horse owners (blue), yard owners (green), and veterinarians (beige) giving advice, referring people to other information sources or both when asked for diet information (χ^2^ = 30.99, df = 4, *p* < 0.001). (**c**) Percentage of horse owners (blue), yard owners (green), and veterinarians (beige) giving advice, referring people to other information sources or both when asked for management information (χ^2^ = 22.48, df = 4, *p* < 0.001). (**d**) Percentage of horse owners (blue), yard owners (green), and veterinarians (beige) giving advice, referring people to other information sources or both when asked for training information (χ^2^ = 16.27, df = 4, *p* = 0.003). (**e**) Percentage of horse owners (blue), yard owners (green), and veterinarians (beige) giving advice, referring people to other information sources or both when asked for behaviour information (χ^2^ = 19.50, df = 4, *p* = 0.001). (**f**) Percentage of horse owners (blue), yard owners (green), and veterinarians (beige) giving advice, referring people to other information sources or both when asked for welfare information (χ^2^ = 30.35, df = 4, *p* < 0.001).

There was a significant difference between horse owners, yard owners and veterinarians on whether they gave advice, referred people to other sources of information or did both in relation to questions on health, diet, management, training, behavior, and welfare (Figure 3a–f).

### 4.4. Awareness of the Defra Code of Practice (DCP)

Overall, 60.6% (366/604) of respondents had heard about the DCP. However, this differed significantly between horse owners, yard owners and veterinarians (χ^2^ = 20.30, df = 2, *p* < 0.001) with 85.3% (29/34) of veterinarian respondents knowing about the code, 77.8% (49/63) of yard owners, but only 56.8% (288/507) of horse owners.

Similarly, there was a significant difference between the percentage of horse owners, yard owners and veterinarian respondents that had read the DCP (χ^2^ = 23.17, df = 2, *p* < 0.001). Of those respondents who had heard of the DCP, 93.3% (28/30) of the veterinarian respondents had read it, 81.6% (40/49) of yard owners but only 57.4% (167/291) of horse owners.

A third of respondents (33.4% 200/598) had looked at other equine welfare guidelines or codes of practice as well as, or instead of, the DCP and this differed significantly between the three respondent groups (χ^2^ = 23.35, df = 2, *p* < 0.001). Over half (57.4% 35/61) of yard owners had looked at other equine welfare guidelines or recommendations, compared to just under half of the veterinarian respondents (48.5% 16/33) and under a third of horse owners (29.6% 149/504). The other guidelines mentioned by respondents included those published by the British Horse Society, welfare charities including the RSPCA and World Horse Welfare, the British Equine Veterinary Association and the Animal Welfare Act 2006. 

An open-ended question was used to explore the respondents’ perceptions of the efficacy of the DCP. Qualitative analysis identified common themes within the responses, and these are summarized here. All responses were analysed regardless of how the respondent had answered the earlier closed questions on the DCP. Overall, 23.3% (94/403) of respondents suggested that the DCP is unlikely to be read by most equine stakeholders and that not many know about it. In their response to this question, 23.1% (93/403) of respondents said they had not read it themselves. Barriers for engagement included poor accessibility and exposure (19% 77/403), and the perception that the content was too vague (3.2% 13/403), too long (2.5% 10/403), too dry (2.7% 11/403), too official (1.0% 4/403), and perceived to be targeted at novice horse owners (1.5% 6/403).

Some respondents were deterred by the focus on minimum standards rather than best practice (15.2% 61/403) and the association with DEFRA (11.2% 45/403). Respondents suggested that engagement could be improved through greater use of social media to promote the DCP (4.5% 18/403) and by publishing the DCP through the British Horse Society (3.7% 15/403), a body that was more widely recognised and trusted by the equine sector, particularly horse owners, than DEFRA.

### 4.5. Communication Preferences

**Figure 4:**
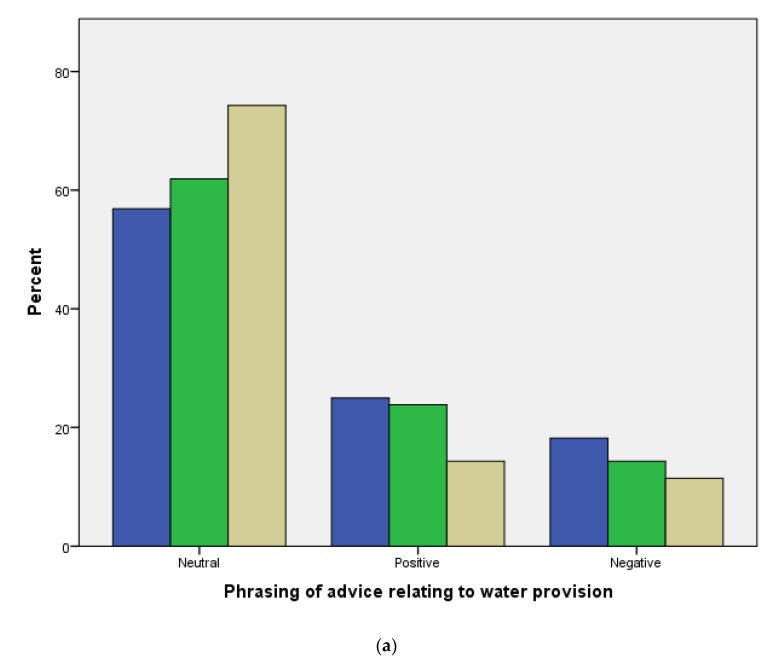

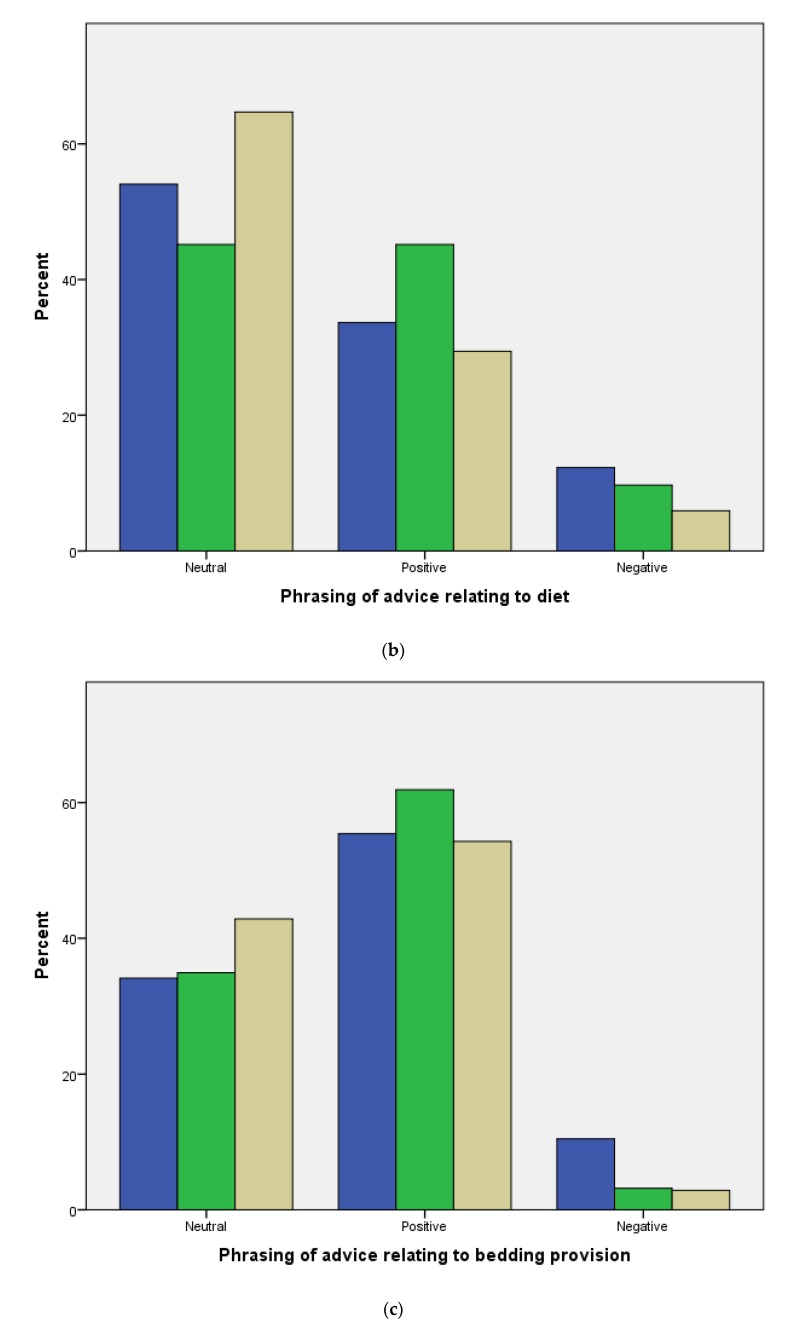
(**a**) Percentage of horse owners (blue), yard owners (green), and veterinarians (beige) selecting their preference for neutrally, positively, and negatively worded statements giving advice on water provision (*p* > 0.05). (**b**) Percentage of horse owners (blue), yard owners (green), and veterinarians (beige) selecting their preference for neutrally, positively, and negatively worded statements giving advice on diet (*p* > 0.05). (**c**) Percentage of horse owners (blue), yard owners (green), and veterinarians (beige) selecting their preference for neutrally, positively, and negatively worded statements giving advice on bedding provision (*p* > 0.05).

Respondents were asked to select which advisory statement they preferred for three subject area, water provision, diet, and bedding. Overall, nearly half of the respondents (49% 886/1804) favoured neutrally worded statements, 38% (689/1804) positive statements and 13% (229/1804) negative statements. There were no significant differences between horse owners, yard owners and veterinarian communication preferences (Figure 4a–c).

Respondents were asked what information they would need to make them more likely to try a new method of management for their horse. The majority of respondents (85.7% 508/593) felt that including information showing the benefits of using the new method would encourage them to try it and 42.2% (250/593) said showing the negative effects of not applying this method should be included. Only 2.9% (17/593) of respondents said that no additional information would be needed, just a description of the method. Three quarters of respondents (76.9% 456/593) said that advice on how to implement the method in practice would encourage them to try it, while 27.0% (160/593) of respondents stated that including examples of how specific horses have suffered from other methods would motivate them to try a new method of management.

There was a significant difference between horse owners, yard owners, and veterinarian respondents on whether evidence taken from scientific papers should be included alongside a description of a new method to make it more likely for them to try it (χ^2^ = 10.326, df = 2, *p* = 0.005). More veterinarians (87.1% 27/31) than horse (64.2% 321/500) and livery yard owners (53.2% 33/62) believed that evidence taken from scientific papers should be included.

When it came to the inclusion of more positive information, there was a significant difference between the three respondent groups (χ^2^ = 8.646, df = 2, *p* = 0.012), with 64.6% (323/500) of horse owners and 59.7% (37/62) of yard owners stating that they would like to see examples of specific horses thriving under the new method of management to motivate them to try it, compared to only 38.7% (12/31) of veterinarians.

## 5. Discussion

The findings of this relatively small UK survey provide a valuable insight into the information-seeking behaviour and communication preferences of three key equine stakeholder groups; horse owners, veterinarians, and yard owners. While the small sample of yard owners and equine veterinarians obtained by the survey may limit the generalizability of the study findings, the identified differences between stakeholder groups in their information-seeking behavior, knowledge of the DCP, and communication preferences all warrant more detailed investigation. The study findings are discussed below in relation to how they can inform recommendations to facilitate more effective and impactful equine welfare communication strategies in future.

The information sources used by respondents varied with stakeholder group and the subject on which advice was sought. While the age difference identified between stakeholder groups may have influenced information-seeking behavior, the age variation within and between stakeholder groups should be acknowledged as it emphasizes the importance of using multiple channels of communication rather than a one size fits all approach to information dissemination. One significant finding across all stakeholder groups is that people—rather than organisations, printed text, or online resources—are the most often consulted information source. The one exception to this being veterinarians use of scientific journals. In the case of riding instructors, animal behaviourists and veterinarians the individuals consulted may be perceived as experts in their fields; however, the key role that horse owners play as an information source for other owners should not be downplayed.

It could be hypothesised that these findings may be a natural progression of the background of each stakeholder group in the equine community. Veterinarians will mostly have learnt about horses through their scientific degrees in which they had to read scientific journals. Therefore, it is perhaps unsurprising that they then have a preference towards scientific journals as a source of information. Moreover, horse owners will have generally initially learnt about horses from other horse owners, riding instructors and yard owners and therefore are much more likely to continue to use them as a source of advice over scientific journals. Additionally, the ease of access in which sources of information can be sought are also a possible explanation to the differences in information source preferences between the groups. Access to scientific journals is often restricted to those who pay a subscription fee; consequently, they may be more easily available to veterinarians who may have access through their profession, than they are to horse or yard owners. Horse owners were much more likely to choose other horse owners as a source of information whereas yard owners and especially veterinarians did not choose horse owners as regularly as part of their top three most likely sources of information to use. This was not an option covered in the previous studies [19,20]. These findings suggest that horse owners have a large influence on each other’s knowledge, highlighting how easily social norms can shape behaviour as has been discussed by other authors [21]. Similarly, yard owners are much more likely to choose yard owners, and veterinarians are much more likely to pick veterinarians than horse owners and yard owners are. There is no comparable data for yard owners, however, this provides a useful starting point, although the field would benefit from further studies. These results suggest that there is perhaps an attitude of trusting others in the same situation with the same training as you. Indeed, in a study on communication about climate change, Nerlich et al. [22] found that citizens preferred communication between each other rather than communication from the government. The results in this study suggest a similar preference. This causes complications in some respects in terms of communicating guidelines; however, it does also suggest that if some of the horse owners are convinced by guidelines and recommendations, then it is likely that the equine community will diffuse this knowledge between themselves.

Unlike in the previous studies, online searches were a relatively popular source of information. In contrast, Hockenhull and Creighton’s study [20] in the UK and Visser and Van Wijk-Jansen’s study [19] in the Netherlands revealed that the internet was not found to be a primary source of information despite owners’ online presence. However, as Dumitru et al. [23] discovered in human medical health, these findings suggest a similar hypothesis that participants may be using the internet to find their bearings on a subject but do not then use it as their main way of delving deeper into a topic. Additionally, given that the data from these studies were collected between 2006 and 2008, these different results may simply reflect a more up to date picture of how searching behaviour has evolved over the last decade with the advance in technology recording an increase in the used of the internet by horse owners as predicted in these previous studies [20,24]. Many charities use the internet as their main platform to address welfare issues. Hockenhull and Creighton’s study [20] queried this and explored whether the internet is the best method to relay information to horse owners. Visser and Van Wijk-Jansen [19] explained that this knowledge could be used to put into place more effective communications with horse owners. Therefore, this study suggests that using the internet could be an effective way of disseminating knowledge. Using mass-media communications has been found to be a very efficient means of transferring information and increasing awareness [25]. It also suggests that giving professionals website links to share with horse owners might be a good way of getting the information communicated effectively to horse owners. However, it is important to note that while the use of internet for online searches was popular, the use of the internet for online forums was not. This could be explained by the fact that horse owners are more likely to ask people on their yard at the time of the problem rather than trusting the advice of strangers. In contrast, many participants suggested that social media should be used more advantageously to publicise guidelines in future. Indeed, Moorhead et al. [26] found in a public health study that the use of social media was an effective way of widening access to health information and increased the accessibility of the information. Lovejoy and Saxton’s study [27] revealed that the use of social media such as Twitter by non-profit organisations has been more successful at engaging their stakeholders than the use of traditional websites ever was, promising an increase in the public’s engagement. However, due to the way participants were recruited, there is likely to be a social media bias in this sample. Nevertheless, the idea of using multiple channels to communicate information such as through the use of the websites and social media and through professionals is supported by Martinson et al.’s [28] and Sullivan’s findings [29], which concluded that horse owners looking for information prefer to receive information through multiple channels.

Veterinarians were found to search for information more regularly than the other stakeholder groups. This could reflect their ongoing professional requirement to stay abreast of recent developments in the field, rather than true information-seeking behavior. However, given the small number of veterinarians sampled and the lack of detail generated this is purely speculative. Differentiating between the underlying motivations for information-seeking behavior and how this may impact on the sources consulted warrants further investigation.

Both veterinarians and yard owners were found to most commonly give advice themselves rather than refer owners to other sources of information or do both. All the veterinarians sampled and about three quarters of yard owners acknowledged that they had an important role in passing on new research information to horse owner. As data were not collected on how frequently stakeholders were asked for advice, it is difficult to ascertain the significance of advice-giving behavior. However, other studies also recognise the role of veterinarians in informing clients of best practice [30]. In their recent book, Bekoff and Pierce [31], encapsulate this idea perfectly with their statement that “vets are really the linchpin for animal welfare” pp. 136. In our study sample only 11% of veterinarians and 16% of yard owners said that they would refer owners to sources of information other than themselves. Further research to understand the reasons behind this would be beneficial. Perhaps they feel that their knowledge of the subject is complete and up to date; or perhaps they are not aware of a reliable information source to direct people to. Equine vets are not alone in this. In a recent study of online searching for pet health information, UK pet owners reported that their veterinarian never recommended specific websites that they could go to look for additional information, but over 90% of these owners said they would visit specific websites if they had been recommended by their vet [32]. Given the apparent preference for organisations to make information and guidelines accessible via the internet, having specific sites recommended by veterinarians may help people to direct their online searches to more reliable resources.

Therefore, trying to encourage professionals to share this information is also key to effectively communicating new research to the equine public. This means that professionals and paraprofessionals such as veterinarians, riding instructors, animal behaviourists, and yard owners should be targeted with the new scientific information, provided support in understanding it, and encouraged to share their information with many horse owners. Lam et al. [33] and Horseman et al. [13,34] emphasise that veterinarians and other equine welfare professionals need to take advantage of current knowledge about human behaviour change to encourage owners to modify their behaviour to ensure the best welfare for their horse. Bard et al. [35] found that veterinarians’ communication with clients was mostly based on a directive style using the paternalism approach with the client having a passive role. However, Bard et al. [35] found that this communication method was unlikely to encourage clients towards behavioural change and more likely to lead to psychological reactance. Lam et al. [33] and Bard et al. [35] suggested that veterinarians need to learn to communicate effectively with the client, by personalizing their response according to their client and building an emotional rapport with the client. Lam et al. [33] also suggested that there was a lack of transfer of information from veterinarians to horse owners and explored this issue further in their review. Veterinarians are recognised as trusted information sources within [36,37] and outside [32,38,39] the equine community. Capitalising on this, by disseminating recent equine welfare research findings to veterinarians to ensure their knowledge is up to date, will facilitate their role as a highly effective conduit of evidence-based advice for horse owners. Research has explored the barriers that equine veterinarians perceive as hindering their uptake of knowledge, e.g., lack of time, difficulty accessing published articles, and identified that concise, visual presentation of information will optimise uptake [40]. Such research can be used to improve veterinarians’ access to and engagement with scientific research and thereby their ability to pass this information on to horse owners.

The majority of survey respondents from all three stakeholder groups were found to know about the DCP. However, many respondents had not read it, particularly in the horse owners’ group where only a third reported actually having read the DCP. This suggests that although the guidelines have reached a large majority of veterinarians and yard owners sampled in this study, this knowledge is again not getting through to enough horse owners or that they are simply not engaging with it. Reasons why this may be the case suggested by respondents included the length, the focus on minimum standards rather than best practice and the association with DEFRA as the issuing body. This is concerning as several respondents even went as far as explaining that they would not read these guidelines as they were associated with DEFRA e.g., “Wouldn’t read DEFRA. Associate it with farm animals and we all know what appalling lives they have.” DEFRA is not always seen as a help but as a “rule-churning government bother” and there was “no faith in DEFRA at all” from some participants. This may reflect the “We-Them accentuation” explored by Castro and Batel [41] which refers to the distinction between experts and citizens, where the former decide how things should be done. This is not conducive to effective behaviour change and either needs to be rectified by changing the image of DEFRA in the equine community or by making sure that guidelines and knowledge are distributed through other organisations that are already affiliated with the equine community and are generally trusted by this population. The respondents suggested that the British Horse Society might provide such an avenue for dissemination. While not every equine stakeholder will preferentially choose the BHS as their go-to information resource, it may be useful to use their established name and communication routes to as one of multiple conduits to produce and publicise equine welfare guidelines to optimise engagement and uptake by equine stakeholders. The findings suggest that the DCP could have more impact by promoting best practice rather than detailing minimum requirements for equine welfare. This is an encouraging finding and one that should certainly be investigated further.

Another important consideration is the language and tone used in equine welfare communications. Indeed, it is widely recognised that human perception of information is influenced more by how something is said than it is by exactly what is said [42]. Consequently, the way that information is framed can profoundly impact its uptake. Interestingly, respondents showed a preference for information presented in a neutral way with no emphasis on why not doing something may be bad (negative) or why doing it would be good (positive). Information worded in a positive way was preferred by 38% of respondents, with only 13% preferring the negatively worded statements suggesting that this is the least effective tone of communication to use. While our findings may have been affected by the lack of randomization in the order in which the statements were presented (all respondents in all stakeholder groups received the statements in the same order), our findings were in line with the recommendation by Horseman et al. [13]. The authors suggest avoiding negative phraseology to prevent readers becoming defensive and associating their negative attitudes with the distributed information. Given that only 13% chose negative statements, this suggests that this would be the least effective tone of communication to use, as Horseman et al. [13] suggested. Information that elicits discomfort maybe avoided by the very people you are trying to reach [43]. Weiman [44], discussed the use of fear-arousing messages to create behaviour change or adherence and concluded that although the literature suggested that this technique would have an effect, the result is neither consistent nor predictable. Consequently, positive and neutral terminology would be most appropriate to communicate effectively. This correlates with Horseman et al.’s findings [45]. Indeed, their study found that the interviewees would distance themselves from being associated with poor welfare. Horseman et al. [13] suggested using more positive and fully understood terms should be considered to avoid defensive negative attitudes. Additionally, these findings also suggest that charities should possibly avoid using much negativity in their campaigns and adverts and change their methods to a more encouraging approach.

When it came to what explanations should be included in an equine welfare communication, only 42.2% of respondents said that information showing the negative effects of not doing something should be included and 27 % of respondents said that examples of specific horses that have suffered from not doing something should be included. This further emphasises that exposing the negative effects or illustrating facts with negative examples may not be beneficial in engaging equine stakeholders and eliciting behaviour change. Less than 3% of respondents said that communications should simply state the facts with no explanations given, suggesting that although the neutral language was preferred by respondents, there is a need for the information given to be explained and put into context.

By far the most preferred explanations to be included in equine welfare communications were positive in nature, with 85.7% of respondents wanting information on the benefits of following the advice. The majority of veterinarians and yard owners also thought that examples of specific horses that had thrived with this new method should be included whereas the majority of owners did not select this option. This may indicate that veterinarians and yard owners are keen to showcase evidence to break the social norms, but horse owners are more reluctant to. This is potentially because horse owners represent a very broad group and therefore individually, they may feel that the specific case is not relevant to them. At a practical level, respondents were keen in see advice on how to implement this information in practice. The fact that how to implement methods is so highly regarded may highlight a barrier with the current guidelines. Indeed, Castro and Batel [41] suggested that for social change to occur, the changes need to be explained in context and information given of how the changes would work in practice. Over half the participants also suggested that evidence taken from scientific papers should be included. However, there was a significant difference between horse owners, yard owners, and veterinarians in the percentage that thought this should be included with veterinarians being most keen to have this included (87.1%), followed by yard owners (64.2%) followed by horse owners (53.2%). Contrary to popular opinion, Engdahl and Lidskog [46] suggest that it is not a fear or distrust of scientific knowledge but rather the fact that scientists underestimate the abilities of the public and that they therefore make less detailed reports, which are harder for the public to engage with and relate to their knowledge. Indeed, Nisbet and Scheufele [47] suggested that tailored information should to promote behaviour change given the technological advances, which make it possible with relative ease. Despite some equestrians voicing negative beliefs about science [48], within the equine community we are seeing increasing engagement with scientific research through media channels. Many printed and online lay publications contain synopses of recently published research, and citizen science approaches are gaining popularity with equine researchers providing another platform through which the equine community can engage with science, e.g., [49].

## 6. Conclusions

To facilitate human behaviour change leading to equine welfare improvement, evidence-based knowledge needs to be made available to those who can benefit from it. Multiple routes of knowledge transmission exist and for information dissemination to be effective we need to understand how these are used by relevant stakeholders. Our findings suggest that we need to move away from our traditional dissemination routes to promote best practice rather basic standards and to consider the level of trust in the issuing organisation to maximise the effectiveness of our communication. Greater emphasis should be placed on positive language and the inclusion of examples, as well as practical details of how changes can be implemented. Industry professionals, particularly veterinarians, have an important role to play in knowledge transfer and the dissemination of research findings to horse owners.

## Supplementary Materials

The following are available online at https://www.mdpi.com/2076-2615/10/1/21/s1, Survey S1: Implementing Equine Research.
