# Supplementary material for: Optimising the Efficacy of Equine Welfare Communications: Do Equine Stakeholders Differ in Their Information-Seeking Behaviour and Communication Preferences?
Source: Animals (Basel). 2019 Dec 20;10(1):21. doi: 10.3390/ani10010021 (PMC7022754; doi:10.3390/ani10010021)
Supplement: Supplementary file 1 [file animals-10-00021-s001.pdf]

Implementing Equine Research

Hello!

Thank you very much for considering filling out my survey! The data that I am trying to collect is key to a very exciting piece of research that I am doing with the University of Bristol's Animal Welfare and Behaviour group. I am very passionate about this research and truly believe that this information could help many horses lead a happier life, but to do this I need your thoughts on the topic.

I am trying to find out how equine information can be communicated more effectively to horse owners to ensure the well-being of their horses. To do this I have made an online survey. If you live in the UK and own or look after a horse or are an equine veterinarian or yard owner and would like to take part please read the following and then click on the 'Next' button below.

This online survey consists of seven sections about your equine background, your thoughts on equine management, how you seek information and how you would most like information to be communicated to you. The survey takes approximately 10-15 minutes to complete.

I would like to encourage as many people as possible to complete this survey to help us to fully understand how best to communicate information with horse owners. Your answers are extremely important to help us better understand this topic; however, your participation in this survey is completely voluntary. Your answers, in all cases, will be treated with complete confidentiality, and I cannot trace anybody back from the answers given here. You may choose to stop participating in the survey at any time. If you would like to withdraw once you have submitted the survey you can, just email me. By clicking on the survey link below, you are indicating that you understand and consent to the terms above and agree to participate in this survey.

The results generated will be used for my research project. If you are interested in the results, you can email me and I will be happy to share my findings.

If you have concerns or questions about this study, please do not hesitate to contact me.

Your participation is greatly appreciated. Thank you very much for your time!

Persephone Pickering

pp14228@my.bristol.ac.uk

University of Bristol.

About you

We are interested in hearing from different groups of horse people - owners/carers, yard owners and veterinarians to understand their different perspectives.

Which survey would you like to take? ✱ *Required*

- ○ Horse owner / Loan a horse
- ○ Yard owner
- ○ Veterinarian

About you

What age category do you belong to?

- ○ 16-25 years
- ○ 26-45 years
- ○ 46-65 years
- ○ 65+ years

What is the **first** part of your postcode?

Your answer should be no more than 4 characters long.

How did you **first** get involved with horses? Please tick **all** that apply.

- ☐ Through family and friends with an equine background
- ☐ Through family and friends with an agricultural background
- ☐ Through riding lessons at a riding school
- ☐ Through Pony Club
- ☐ Through owning or loaning a horse with no previous contact with horses
- ☐ Through working on a yard
- ☐ Other

If you selected Other, please specify:

Do you own your own horse?

- Yes
- No

How long have you run a livery yard?

- Under year
- 1-5 years
- 6-10 years
- 11-20 years
- 21+ years

Select the people you are in **any** contact with, either **personally** or **professionally.** Please **tick** all that apply.

- Animal Behaviour or Welfare researcher
- Animal Welfare charity worker
- Animal Welfare charity
- Animal Behaviour or Welfare tutor
- Veterinarian
- Farrier
- Yard owner
- Equestrian organisation (eg. BHS)

About you

What age category do you belong to?

- ◯ 16-25 years
- ◯ 26-45 years
- ◯ 46-65 years
- ◯ 65+ years

What is the **first** part of your postcode?

Your answer should be no more than 4 characters long.

How did you **first** get involved with horses? Please tick **all** that apply.

- ☐ Through family and friends with an equine background
- ☐ Through family and friends with an agricultural background
- ☐ Through riding lessons at a riding school
- ☐ Through Pony Club
- ☐ Through owning or loaning a horse with no previous contact with horses
- ☐ Through your veterinary training
- ☐ Other

If you selected Other, please specify:

Do you own your own horse?

- Yes
- No

How long have you had experience with horses as a veterinarian?

- Under year
- 1-5 years
- 6-10 years
- 11-20 years
- 21+ years

Select the people you are in **any** contact with, either **personally** or **professionally**. Please tick **all** that apply.

- Animal Behaviour or Welfare researcher
- Animal Welfare charity worker
- Animal Welfare charity
- Animal Behaviour or Welfare tutor
- Veterinarian
- Yard owner
- Farrier
- Equestrian organisation (eg. BHS)

About you

What age category do you belong to?

- ○ 16-25 years
- ○ 26-45 years
- ○ 46-65 years
- ○ 65+ years

What is the **first** part of your postcode?

Your answer should be no more than 4 characters long.

How did you **first** get involved with horses? Please tick **all** that apply.

- ☐ Through family and friends with an equine background
- ☐ Through family and friends with an agricultural background
- ☐ Through riding lessons at a riding school
- ☐ Through Pony Club
- ☐ Through owning or loaning a horse with no previous contact with horses
- ☐ Other

If you selected Other, please specify:

Do you own your own horse?

- ○ Yes

- No
- Other

If you selected Other, please specify:

How long have you had experience with horses?

- Under year
- 1-5 years
- 6-10 years
- 11-20 years
- 21+ years

Select the people you are in **any** contact with, either **personally** or **professionally**. Please tick **all** that apply.

- Animal Behaviour or Welfare researcher
- Animal Welfare charity worker
- Animal Welfare charity
- Animal Behaviour or Welfare tutor
- Veterinarian
- Yard owner
- Farrier
- Equestrian organisation (eg. BHS)

Your thoughts on equine management

Please tick the option that you feel is the **most accurate** statement for horses generally in each section below.

Bedding choices:

- ○ Rubber mats are a good bedding material on their own for a horse
- ○ Rubber mats alone are not sufficient bedding for a horse
- ○ Horses do not need bedding in their stables

Social lives:

- ○ Housing horses individually is better for horses so long as they have contact with their owner
- ○ Housing horses with other horses is best for their welfare
- ○ Restricted social contact (muzzle/head) is better for their welfare than full social contact (whole body)

Riding:

- ○ Horses prefer to be ridden alone by their owner
- ○ Horses prefer to be ridden with other horses present as well as their owner
- ○ For the horse, being ridden is a good replacement for turn-out time

Horse friendships:

- ○ Horses form a close bond with a specific horse
- ○ Horses form close bonds with several horses
- ○ Horses have no preference for which horses they spend time with

Use of rugs:

- All horses need to be covered in winter with a rug to keep warm
- The need for a rug depends on the individual horse's coat and condition
- Rugs need to be used when the temperature drops below 5 degrees centigrade whatever time of year it is

Horse behaviour:

- Weaving is a method of communication between horses in different stables
- Weaving is a sign of frustration and should be fixed by preventing the action (eg. anti-weave grill)
- Weaving is copied from other animals that have poor welfare
- Weaving is a sign of frustration and should be stopped by finding the cause of the behaviour

Stabling:

- All horses need to be stabled for their safety
- Horses do not necessarily need to be stabled
- All horses enjoy being stabled

Feeding choices:

- Access to ad lib (freely available) forage is the best feeding strategy for a horse unless it presents a health risk
- Feeding concentrates is the best strategy as it ensures the horse gets all the vital nutrition it needs
- Horses should have periods of time where food is not accessible between feeding

Rewarding horses:

- ○ Patting is naturally rewarding for horses
- ○ Scratching the withers is naturally rewarding for horses
- ○ Verbal praise is naturally rewarding for horses

Your thoughts on equine management

Please tick the option that you feel is the **most accurate** statement for horses generally in each section below:

Bedding choices:

- Rubber mats are a good bedding material on their own for a horse
- Rubber mats alone are not sufficient bedding for a horse
- Horses do not need bedding in their stables

Social lives:

- Housing horses individually is better for horses so long as they have contact with their owner
- Housing horses with other horses is best for their welfare
- Restricted social contact (muzzle/head) is better for their welfare than full social contact (whole body)

Riding:

- Horses prefer to be ridden alone by their owner
- Horses prefer to be ridden with other horses present as well as their owner
- For the horse, being ridden is a good replacement for turn-out time

Horse friendships:

- Horses form a close bond with a specific horse
- Horses form close bonds with several horses
- Horses have no preference for which horses they spend time with

Use of rugs:

- All horses need to be covered in winter with a rug to keep warm
- The need for a rug depends on the individual horse's coat and condition
- Rugs need to be used when the temperature drops below 5 degrees centigrade whatever time of year it is

Horse behaviour:

- Weaving is a method of communication between horses in different stables
- Weaving is a sign of frustration and should be fixed by preventing the action (eg. anti-weave grill)
- Weaving is copied from other animals that have poor welfare
- Weaving is a sign of frustration and should be stopped by finding the cause of the behaviour

Stabling:

- All horses need to be stabled for their safety
- Horses do not necessarily need to be stabled
- All horses enjoy being stabled

Feeding choices:

- Access to ad lib (freely available) forage is the best feeding strategy for a horse unless it presents a health risk
- Feeding concentrates is the best strategy as it ensures the horse gets all the vital nutrition it needs
- Horses should have periods of time where food is not accessible between feeding

Rewarding horses:

- ◯ Patting is naturally rewarding for horses
- ◯ Scratching the withers is naturally rewarding for horses
- ◯ Verbal praise is naturally rewarding for horses

Your thoughts on equine management

Please tick the option that you feel is the **most accurate** statement for horses generally in each section below:

Bedding choices:

- Rubber mats are a good bedding material on their own for a horse
- Rubber mats alone are not sufficient bedding for a horse
- Horses do not need bedding in their stables

Social lives:

- Housing horses individually is better for horses so long as they have contact with their owner
- Housing horses with other horses is best for their welfare
- Restricted social contact (muzzle/head) is better for their welfare than full social contact (whole body)

Riding:

- Horses prefer to be ridden alone by their owner
- Horses prefer to be ridden with other horses present as well as their owner
- For the horse, being ridden is a good replacement for turn-out time

Horse friendships:

- Horses form a close bond with a specific horse
- Horses form close bonds with several horses
- Horses have no preference for which horses they spend time with

Use of rugs:

- All horses need to be covered in winter with a rug to keep warm
- The need for a rug depends on the individual horses coat and condition
- Rugs need to be used when the temperature drops below 5 degrees centigrade whatever time of year it is

Horse behaviour:

- Weaving is a method of communication between horses in different stables
- Weaving is a sign of frustration and should be fixed by preventing the action (eg. anti-weave grill)
- Weaving is copied from other animals that have poor welfare
- Weaving is a sign of frustration and should be stopped by finding the cause of the behaviour

Stabling:

- All horses need to be stabled for their safety
- Horses do not necessarily need to be stabled
- All horses enjoy being stabled

Feeding choices:

- Access to ad lib forage (freely available) is the best feeding strategy for a horse unless it presents a health risk
- Feeding concentrates is the best strategy as it ensures the horse gets all the vital nutrition it needs
- Horses should have periods of time where food is not accessible between feeding

Rewarding horses:

- Patting is naturally rewarding for horses
- Scratching the withers is naturally rewarding for horses
- Verbal praise is naturally rewarding for horses

How do you find equine information?

We are interested in which sources of information you use to find **reliable** information that you **trust** about different horse-related issues.

For each scenario, please select the **top three** information sources that you use **most often** to get this **reliable** information and the information source you are **least likely** to consider using in this scenario.

If a horse seemed unwell and you did not know what was wrong, where would you be most likely to go for reliable information you would follow?

	Source of information	Why would you choose this source?
1st most likely to go to	Please select	
2nd most likely to go to	Please select	
3rd most likely to go to	Please select	
Least likely to go to	Please select	

If you had concerns about a horse's diet and feeding regime, where would you be most likely to go for reliable information you would follow?

	Source of information	Why would you choose this source?

1st most likely to go to	Please select	
2nd most likely to go to	Please select	
3rd most likely to go to	Please select	
Least likely to go to	Please select	

If you were concerned about the way a horse is housed or managed (eg. stabled or in a field), where would you be most likely to go for reliable information you would follow?

	Source of information	Why would you choose this source?
1st most likely to go to	Please select	
2nd most likely to go to	Please select	
3rd most likely to go to	Please select	

Least likely to go to	Please select	

If you wanted some advice on a horse's training, where would you be most likely to go for reliable information you would follow?

	Source of information	Why would you choose this source?
1st most likely to go to	Please select	
2nd most likely to go to	Please select	
3rd most likely to go to	Please select	
Least likely to go to	Please select	

If you were concerned about a horse's behaviour in some way, where would you be most likely to go for reliable information you would follow?

	Source of information	Why would you choose this source?

1st most likely to go to	Please select	
2nd most likely to go to	Please select	
3rd most likely to go to	Please select	
Least likely to go to	Please select	

If you wanted to find out how best to look after a horse to ensure their physical and mental well-being, where would you be most likely to go for reliable information you would follow?

	Source of information	Why would you choose this source?
1st most likely to go to	Please select	
2nd most likely to go to	Please select	
3rd most likely to go to	Please select	

Least likely to go to	Please select	

Are there any other sources of information you would be likely to use that were not mentioned?

How frequently on average do you search for equine information?

- ☐ Weekly
- ☐ Monthly
- ☐ Several times a year
- ☐ Once a year
- ☐ Hardly ever
- ☐ Never

What type of information do you have trouble finding?

Do horse owners ask **you** for **advice** on any of the following areas? If so, do you give them advice **yourself** or **refer them** on to other sources of information? Please tick **all** those that apply:

How do you give information?

	Give advice myself	Refer to other sources of information	If you refer them to another source of information, where do you refer them?
Equine health	☐	☐	
Equine nutrition	☐	☐	
Equine housing	☐	☐	
Equine training	☐	☐	
Equine behaviour	☐	☐	
Equine welfare	☐	☐	

How do you find equine information?

We are interested in which sources you use to find **reliable** information that you **trust** about different horse-related issues.

For each scenario, please select the **top three** information sources that you use **most often** to get this **reliable** information and the information source you are **least likely** to consider using in this scenario.

If a horse seemed unwell and you did not know what was wrong, where would you be most likely to go for reliable information you would follow?

	Source of information	Why would you choose this source?
1st most likely to go to	Please select	
2nd most likely to go to	Please select	
3rd most likely to go to	Please select	
Least likely to go to	Please select	

If you had concerns about a horse's diet and feeding regime, where would you be most likely to go for reliable information you would follow?

	Source of information	Why would you choose this source?

1st most likely to go to	Please select	
2nd most likely to go to	Please select	
3rd most likely to go to	Please select	
Least likely to go to	Please select	

If you were concerned about the way a horse is housed or managed (eg. stabled or in a field), where would you be most likely to go for reliable information you would follow?

	Source of information	Why would you choose this source?
1st most likely to go to	Please select	
2nd most likely to go to	Please select	
3rd most likely to go to	Please select	

Least likely to go to	Please select	

If you wanted some advice on a horse's training, where would you be most likely to go for reliable information you would follow?

	Source of information	Why would you choose this source?
1st most likely to go to	Please select	
2nd most likely to go to	Please select	
3rd most likely to go to	Please select	
Least likely to go to	Please select	

If you were concerned about a horse's behaviour in some way, where would you be most likely to go for reliable information you would follow?

	Source of information	Why would you choose this source?

1st most likely to go to	Please select	
2nd most likely to go to	Please select	
3rd most likely to go to	Please select	
Least likely to go to	Please select	

If you wanted to find out how best to look after a horse to ensure their physical and mental well-being, where would you be most likely to go for reliable information you would follow?

	Source of information	Why would you choose this source?
1st most likely to go to	Please select	
2nd most likely to go to	Please select	
3rd most likely to go to	Please select	

Least likely to go to	Please select	

Are there any other sources of information you would be likely to use that were not mentioned?

How frequently on average do you search for equine information?

- ☐ Weekly
- ☐ Monthly
- ☐ Several times a year
- ☐ Once a year
- ☐ Hardly ever
- ☐ Never

What type of information do you have trouble finding?

Do horse owners ask **you** for **advice** on any of the following areas? If so, do you give them advice **yourself** or **refer them** on to other sources of information? Please tick **all** those that apply:

	How do you give information?

	Give advice myself	Refer to other sources of information	If you refer them to another source of information, where do you refer them?
Equine health	☐	☐	
Equine nutrition	☐	☐	
Equine housing	☐	☐	
Equine training	☐	☐	
Equine behaviour	☐	☐	
Equine welfare	☐	☐	

How do you find equine information?

We are interested in which sources you use to find **reliable** information that you **trust** about different horse-related issues.

For each scenario, please select the **top three** information sources that you use **most often** to get this **reliable** information and the information source you are **least likely** to consider using in this scenario.

If your horse seemed unwell and you did not know what was wrong, where would you be most likely to go for reliable information you would follow?

	Source of information	Why would you choose this source?
1st most likely to go to	Please select	
2nd most likely to go to	Please select	
3rd most likely to go to	Please select	
Least likely to go to	Please select	

If you had concerns about your horse's diet and feeding regime, where would you be most likely to go for reliable information you would follow?

	Source of information	Why would you choose this source?

1st most likely to go to	Please select	
2nd most likely to go to	Please select	
3rd most likely to go to	Please select	
Least likely to go to	Please select	

If you were concerned about the way your horse is housed or managed (eg. stabled or in a field), where would you be most likely to go for reliable information you would follow?

	Source of information	Why would you choose this source?
1st most likely to go to	Please select	
2nd most likely to go to	Please select	
3rd most likely to go to	Please select	

Least likely to go to	Please select	

If you wanted some advice on your horse's training, where would you be most likely to go for reliable information you would follow?

	Source of information	Why would you choose this source?
1st most likely to go to	Please select	
2nd most likely to go to	Please select	
3rd most likely to go to	Please select	
Least likely to go to	Please select	

If you were concerned about your horse's behaviour in some way, where would you be most likely to go for reliable information you would follow?

	Source of information	Why would you choose this source?

1st most likely to go to	Please select	
2nd most likely to go to	Please select	
3rd most likely to go to	Please select	
Least likely to go to	Please select	

If you wanted to find out how best to look after your horse to ensure their physical and mental well-being, where would you be most likely to go for reliable information you would follow?

	Source of information	Why would you choose this source?
1st most likely to go to	Please select	
2nd most likely to go to	Please select	
3rd most likely to go to	Please select	

Least likely to go to	Please select	

Are there any other sources of information you would be likely to use that were not mentioned?

How frequently on average do you search for equine information?

- Weekly
- Monthly
- Several times a year
- Once a year
- Hardly ever
- Never

What type of information do you have trouble finding?

Do other horse owners ask **you** for **advice** on any of the following areas? If so, do you give them advice **yourself** or **refer them** on to other sources of information? Please tick **all** those that apply:

	How do you give information?

	Give advice myself	Refer to other sources of information	If you refer them to another source of information, where do you refer them?
Equine health	☐	☐	
Equine nutrition	☐	☐	
Equine housing	☐	☐	
Equine training	☐	☐	
Equine behaviour	☐	☐	
Equine welfare	☐	☐	

Knowledge of welfare guidelines

Do you **know** about the DEFRA Code of Practice for the Welfare of Horses, Ponies, Donkeys and their Hybrids?

- Yes
- No

Have you **read** the DEFRA Code of Practice for the Welfare of Horses, Ponies, Donkeys and their Hybrids?

- Yes
- No

Are there any **other** guidelines or recommendations that you have looked at?

- Yes
- No

If yes, please specify:

What does equine welfare mean to you?

Knowledge of welfare guidelines

Do you **know** about the DEFRA Code of Practice for the Welfare of Horses, Ponies, Donkeys and their Hybrids?

- Yes
- No

Have you **read** the DEFRA Code of Practice for the Welfare of Horses, Ponies, Donkeys and their Hybrids?

- Yes
- No

Are there any **other** guidelines or recommendations that you have looked at?

- Yes
- No

If yes, please specify:

What does equine welfare mean to you?

Knowledge of welfare guidelines

Do you **know** about the DEFRA Code of Practice for the Welfare of Horses, Ponies, Donkeys and their Hybrids?

- Yes
- No

Have you **read** the DEFRA Code of Practice for the Welfare of Horses, Ponies, Donkeys and their Hybrids?

- Yes
- No

Are there any **other** guidelines or recommendations that you have looked at?

- Yes
- No

If yes, please specify:

What does equine welfare mean to you?

Interest in improving equine welfare

Which of the following apply to **you**? Please tick **all** that apply.

- ☐ I think the methods currently used to manage horses are good for their welfare
- ☐ I try to keep up with new ideas on how to keep horses
- ☐ If I see or get told new information on equine welfare I am interested
- ☐ When I see new ideas on how to keep horses I will suggest them to horse owners
- ☐ I actively seek out information for better ways to keep horses
- ☐ I read scientific publications relating to equine welfare
- ☐ I think yard owners have an important role in passing on recent research information to horse owners
- ☐ My yard has rules about how horses can be managed
- ☐ I would consider modifying how my yard is run if there was scientific evidence showing that this would lead to better horse welfare

Interest in improving equine welfare

Which of the following apply to **you?** Please tick **all** that apply.

- ☐ I think the methods currently used to manage horses are good for their welfare
- ☐ I try to keep up with new ideas on how to keep horses
- ☐ If I see or get told new information on equine welfare I am interested
- ☐ When I see new ideas on how to keep horses I will suggest them to horse owners
- ☐ I actively seek out information for better ways to keep horses
- ☐ I read scientific publications relating to equine welfare
- ☐ I think veterinarians have an important role in passing on recent research information to horse owners

Interest in improving equine welfare

Which of the following apply to **you**? Please tick **all** that apply.

- ☐ I think the methods I currently use are good for my horse
- ☐ I try to keep up with new ideas on how to keep horses
- ☐ If I see or get told new information on equine welfare I am interested
- ☐ I actively seek out information for better ways to keep horses
- ☐ I read scientific publications relating to equine welfare
- ☐ I make changes when I see new ideas on how to keep horses

The best way to communicate with horse owners

Guidance for people who own or care for horses can be written in **different** ways.

Please tick the sentence that **you** would be **most likely to follow** for each of these subjects:

Water:

- ○ Horses should have continuous access to a clean supply of fresh water
- ○ Horses without continuous access to a clean supply of fresh water can suffer from dehydration and become seriously ill
- ○ Horses with continuous access to a clean supply of fresh water stay hydrated and healthy

Please explain why:

Diet:

- ○ Horses should be fed an appropriate diet that reflects their needs
- ○ Not feeding an appropriate diet to horses can cause malnutrition and lead to the development of severe health issues
- ○ Feeding your horse an appropriate diet leads to their physical and behavioural well-being

Please explain why:

Bedding:

- All equine accommodation should have appropriate bedding material
- Lack of appropriate bedding material can cause your horse to suffer due to sleep deprivation and injury
- Appropriate bedding material allows your horse to lie down in comfort and provides protection against injury

Please explain why:

If there is evidence of a better method of management for horses, which of the following explanations would **you** want to have **included** to make you **more likely** to try or suggest this **new method**? Please tick **all** that apply.

- Information showing the benefit of this new method
- Information showing the negative effects of not applying this method
- Information stating what method you should use with no need for explanations
- Evidence taken from scientific research papers
- Examples of specific horses that have suffered from other methods
- Examples of specific horses that have thrived with the new method
- Advice on how to implement this method
- Other

If you selected other, please specify:

The best way to communicate with horse owners

Guidance for people who own or care for horses can be written in **different** ways.

Please tick the sentence that **you** would be **most likely to follow** for each of these subjects:

Water:

- Horses should have continuous access to a clean supply of fresh water
- Horses without continuous access to a clean supply of fresh water can suffer from dehydration and become seriously ill
- Horses with continuous access to a clean supply of fresh water stay hydrated and healthy

Please explain why:

Food:

- Horses should be fed an appropriate diet that reflects their needs
- Not feeding an appropriate diet to horses can cause malnutrition and lead to the development of severe health issues
- Feeding your horse an appropriate diet leads to their physical and behavioural well-being

Please explain why:

Bedding:

- All equine accommodation should have appropriate bedding material
- Lack of appropriate bedding material can cause your horse to suffer due to sleep deprivation and injury
- Appropriate bedding material allows your horse to lie down in comfort and provides protection against injury

Please explain why:

If there is evidence of a better method of management for horses, which of the following explanations would **you** want to have **included** to make you **more likely** to try or suggest this **new method**? Please tick **all** that apply.

- Information showing the benefit of this new method
- Information showing the negative effects of not applying this method
- Information stating what method you should use with no need for explanations
- Evidence taken from scientific research papers
- Examples of specific horses that have suffered from other methods
- Examples of specific horses that have thrived with the new method
- Advice on how to implement this method
- Other

If you selected other, please specify:

The best way to communicate with you

Guidance for people who own or care for horses can be written in **different** ways.

Please tick the sentence that **you** would be **most likely to follow** for each of these subjects:

Water:

- ○ Horses should have continuous access to a clean supply of fresh water
- ○ Horses without continuous access to a clean supply of fresh water can suffer from dehydration and become seriously ill
- ○ Horses with continuous access to a clean supply of fresh water stay hydrated and healthy

Please explain why:

Food:

- ○ Horses should be fed an appropriate diet that reflects their needs
- ○ Not feeding an appropriate diet to horses can cause malnutrition and lead to the development of severe health issues
- ○ Feeding your horse an appropriate diet leads to their physical and behavioural well-being

Please explain why:

Bedding:

- All equine accommodation should have appropriate bedding material
- Lack of appropriate bedding material can cause your horse to suffer due to sleep deprivation and injury
- Appropriate bedding material allows your horse to lie down in comfort and provides protection against injury

Please explain why:

If there is evidence of a better method of management for your horse, which of the following explanations would **you** want to have **included** to make you **more likely** to try this **new method**? Please tick **all** that apply.

- [ ] Information showing the benefit of this new method
- [ ] Information showing the negative effects of not applying this method
- [ ] Information stating what method you should use with no need for explanations
- [ ] Evidence taken from scientific research papers
- [ ] Examples of specific horses that have suffered from other methods
- [ ] Examples of specific horses that have thrived with the new method
- [ ] Advice on how to implement this method
- [ ] Other

If you selected other, please specify:

Your experiences

We would like to hear **your thoughts** about the issues covered in this survey.

Please could you answer the following questions in **as much detail possible** to help us understand **your views and experiences.**

Research suggests that yard owners are an **important source of information** for horse owners. What are **your experiences** of that? Could you please share **any examples** of what you have been asked about?

In the past, have you changed the ways in which horses on your yard are kept because of information you have read? Would you be willing to do so in the future? Please could you **give example(s).**

What stops you from treating horses how you would ideally treat them?

Do you think that the 'DEFRA Code of Practice for the Welfare of Horses, Ponies, Donkeys and their Hybrids' and other similar guidelines are a good way of ensuring and promoting good equine welfare? If not, what do **you** think is the **best way** to do so?

Your experiences

We would like to hear **your thoughts** about the issues covered in this survey.

Please could you answer the following questions in **as much detail as possible** to help us understand **your views and experiences**.

Research suggests that veterinarians are an **important source of information** for horse owners. What are **your experiences** of that? Could you please share **any examples** of what you have been asked about?

In the past, have you suggested that horse owners should change how they keep or look after their horse because of information you have read? Would you be willing to do so in the future? Please could you **give example(s)**.

What do you think stops horse owners from treating horses how you would ideally treat them?

Do you think that the DEFRA Code of Practice for the Welfare of Horses, Ponies, Donkeys and their Hybrids and other similar guidelines are a good way of ensuring and promoting good equine welfare? If not, what do **you** think is the **best way** to do so?

Your experiences

We would like to hear **your thoughts** about the issues covered in this survey.

Please could you answer the following questions in **as much detail as possible** to help us understand **your views and experiences.**

In the past, have you changed how you keep or look after horses because of information you have read? Would you be willing to do so in the future? Please could you **give example(s).**

What stops you from treating your horse the way you would ideally treat it?

Do you think that the DEFRA Code of Practice for the Welfare of Horses, Ponies, Donkeys and their Hybrids and other similar guidelines are a good way of ensuring and promoting good equine welfare? If not, what do **you** think is the **best way** to do so?

Thank you!

Many thanks for completing this survey. Please contact me at pp14228@my.bristol.ac.uk if you have any questions or would like to see the results of my survey.

Key for selection options

47.1.a - Source of information

- Riding instructor
- Veterinarian
- Farrier
- Another yard owner
- Equine charity
- Equine physiotherapist
- Animal behaviourist
- Tack shop worker
- Horse owner
- Course I have followed / am following
- Equine magazine
- Internet forums
- Online searches
- Scientific journals
- Books
- DEFRA Code of Practice or similar guidelines
- Other

47.2.a - Source of information

- Riding instructor
- Veterinarian
- Farrier
- Another yard owner
- Equine charity
- Equine physiotherapist
- Animal behaviourist
- Tack shop worker
- Horse owner
- Course I have followed / am following
- Equine magazine
- Internet forums
- Online searches
- Scientific journals

Books
DEFRA Code of Practice or similar guidelines
Other

47.3.a - Source of information

Riding instructor
Veterinarian
Farrier
Another yard owner
Equine charity
Equine physiotherapist
Animal behaviourist
Tack shop worker
Horse owner
Course I have followed / am following
Equine magazine
Internet forums
Online searches
Scientific journals
Books
DEFRA Code of Practice or similar guidelines
Other

47.4.a - Source of information

Riding instructor
Veterinarian
Farrier
Another yard owner
Equine charity
Equine physiotherapist
Animal behaviourist
Tack shop worker
Horse owner
Course I have followed / am following
Equine magazine
Internet forums
Online searches
Scientific journals
Books
DEFRA Code of Practice or similar guidelines
Other

48.1.a - Source of information

- Riding instructor
- Veterinarian
- Farrier
- Another yard owner
- Equine charity
- Equine physiotherapist
- Animal behaviourist
- Tack shop worker
- Horse owner
- Course I have followed / am following
- Equine magazine
- Internet forums
- Online searches
- Scientific journals
- Books
- DEFRA Code of Practice or similar guidelines
- Other

48.2.a - Source of information

- Riding instructor
- Veterinarian
- Farrier
- Another yard owner
- Equine charity
- Equine physiotherapist
- Animal behaviourist
- Tack shop worker
- Horse owner
- Course I have followed / am following
- Equine magazine
- Internet forums
- Online searches
- Scientific journals
- Books
- DEFRA Code of Practice or similar guidelines
- Other

48.3.a - Source of information

- Riding instructor
- Veterinarian
- Farrier
- Another yard owner

Equine charity
Equine physiotherapist
Animal behaviourist
Tack shop worker
Horse owner
Course I have followed / am following
Equine magazine
Internet forums
Online searches
Scientific journals
Books
DEFRA Code of Practice or similar guidelines
Other

48.4.a - Source of information

Riding instructor
Veterinarian
Farrier
Another yard owner
Equine charity
Equine physiotherapist
Animal behaviourist
Tack shop worker
Horse owner
Course I have followed / am following
Equine magazine
Internet forums
Online searches
Scientific journals
Books
DEFRA Code of Practice or similar guidelines
Other

49.1.a - Source of information

Riding instructor
Veterinarian
Farrier
Another yard owner
Equine charity
Equine physiotherapist
Animal behaviourist
Tack shop worker
Horse owner

Course I have followed / am following
Equine magazine
Internet forums
Online searches
Scientific journals
Books
DEFRA Code of Practice or similar guidelines
Other

49.2.a - Source of information

Riding instructor
Veterinarian
Farrier
Another yard owner
Equine charity
Equine physiotherapist
Animal behaviourist
Tack shop worker
Horse owner
Course I have followed / am following
Equine magazine
Internet forums
Online searches
Scientific journals
Books
DEFRA Code of Practice or similar guidelines
Other

49.3.a - Source of information

Riding instructor
Veterinarian
Farrier
Another yard owner
Equine charity
Equine physiotherapist
Animal behaviourist
Tack shop worker
Horse owner
Course I have followed / am following
Equine magazine
Internet forums
Online searches
Scientific journals

Books
DEFRA Code of Practice or similar guidelines
Other

49.4.a - Source of information

Riding instructor
Veterinarian
Farrier
Another yard owner
Equine charity
Equine physiotherapist
Animal behaviourist
Tack shop worker
Horse owner
Course I have followed / am following
Equine magazine
Internet forums
Online searches
Scientific journals
Books
DEFRA Code of Practice or similar guidelines
Other

50.1.a - Source of information

Riding instructor
Veterinarian
Farrier
Another yard owner
Equine charity
Equine physiotherapist
Animal behaviourist
Tack shop worker
Horse owner
Course I have followed / am following
Equine magazine
Internet forums
Online searches
Scientific journals
Books
DEFRA Code of Practice or similar guidelines
Other

50.2.a - Source of information

- Riding instructor
- Veterinarian
- Farrier
- Another yard owner
- Equine charity
- Equine physiotherapist
- Animal behaviourist
- Tack shop worker
- Horse owner
- Course I have followed / am following
- Equine magazine
- Internet forums
- Online searches
- Scientific journals
- Books
- DEFRA Code of Practice or similar guidelines
- Other

50.3.a - Source of information

- Riding instructor
- Veterinarian
- Farrier
- Another yard owner
- Equine charity
- Equine physiotherapist
- Animal behaviourist
- Tack shop worker
- Horse owner
- Course I have followed / am following
- Equine magazine
- Internet forums
- Online searches
- Scientific journals
- Books
- DEFRA Code of Practice or similar guidelines
- Other

50.4.a - Source of information

- Riding instructor
- Veterinarian
- Farrier
- Another yard owner

Equine charity
Equine physiotherapist
Animal behaviourist
Tack shop worker
Horse owner
Course I have followed / am following
Equine magazine
Internet forums
Online searches
Scientific journals
Books
DEFRA Code of Practice or similar guidelines
Other

51.1.a - Source of information

Riding instructor
Veterinarian
Farrier
Another yard owner
Equine charity
Equine physiotherapist
Animal behaviourist
Tack shop worker
Horse owner
Course I have followed / am following
Equine magazine
Internet forums
Online searches
Scientific journals
Books
DEFRA Code of Practice or similar guidelines
Other

51.2.a - Source of information

Riding instructor
Veterinarian
Farrier
Another yard owner
Equine charity
Equine physiotherapist
Animal behaviourist
Tack shop worker
Horse owner

Course I have followed / am following
Equine magazine
Internet forums
Online searches
Scientific journals
Books
DEFRA Code of Practice or similar guidelines
Other

51.3.a - Source of information

Riding instructor
Veterinarian
Farrier
Another yard owner
Equine charity
Equine physiotherapist
Animal behaviourist
Tack shop worker
Horse owner
Course I have followed / am following
Equine magazine
Internet forums
Online searches
Scientific journals
Books
DEFRA Code of Practice or similar guidelines
Other

51.4.a - Source of information

Riding instructor
Veterinarian
Farrier
Another yard owner
Equine charity
Equine physiotherapist
Animal behaviourist
Tack shop worker
Horse owner
Course I have followed / am following
Equine magazine
Internet forums
Online searches
Scientific journals

Books
DEFRA Code of Practice or similar guidelines
Other

52.1.a - Source of information

Riding instructor
Veterinarian
Farrier
Another yard owner
Equine charity
Equine physiotherapist
Animal behaviourist
Tack shop worker
Horse owner
Course I have followed / am following
Equine magazine
Internet forums
Online searches
Scientific journals
Books
DEFRA Code of Practice or similar guidelines
Other

52.2.a - Source of information

Riding instructor
Veterinarian
Farrier
Another yard owner
Equine charity
Equine physiotherapist
Animal behaviourist
Tack shop worker
Horse owner
Course I have followed / am following
Equine magazine
Internet forums
Online searches
Scientific journals
Books
DEFRA Code of Practice or similar guidelines
Other

52.3.a - Source of information

- Riding instructor
- Veterinarian
- Farrier
- Another yard owner
- Equine charity
- Equine physiotherapist
- Animal behaviourist
- Tack shop worker
- Horse owner
- Course I have followed / am following
- Equine magazine
- Internet forums
- Online searches
- Scientific journals
- Books
- DEFRA Code of Practice or similar guidelines
- Other

52.4.a - Source of information

- Riding instructor
- Veterinarian
- Farrier
- Another yard owner
- Equine charity
- Equine physiotherapist
- Animal behaviourist
- Tack shop worker
- Horse owner
- Course I have followed / am following
- Equine magazine
- Internet forums
- Online searches
- Scientific journals
- Books
- DEFRA Code of Practice or similar guidelines
- Other

57.1.a - Source of information

- Riding instructor
- Another veterinarian
- Farrier
- Yard owner

Equine charity
Equine physiotherapist
Animal behaviourist
Tack shop worker
Horse owner
Course I have followed / am following
Equine magazine
Internet forums
Online searches
Scientific journals
Books
DEFRA Code of Practice or similar guidelines
Other

57.2.a - Source of information

Riding instructor
Another veterinarian
Farrier
Yard owner
Equine charity
Equine physiotherapist
Animal behaviourist
Tack shop worker
Horse owner
Course I have followed / am following
Equine magazine
Internet forums
Online searches
Scientific journals
Books
DEFRA Code of Practice or similar guidelines
Other

57.3.a - Source of information

Riding instructor
Another veterinarian
Farrier
Yard owner
Equine charity
Equine physiotherapist
Animal behaviourist
Tack shop worker
Horse owner

Course I have followed / am following
Equine magazine
Internet forums
Online searches
Scientific journals
Books
DEFRA Code of Practice or similar guidelines
Other

57.4.a - Source of information

Riding instructor
Another veterinarian
Farrier
Yard owner
Equine charity
Equine physiotherapist
Animal behaviourist
Tack shop worker
Horse owner
Course I have followed / am following
Equine magazine
Internet forums
Online searches
Scientific journals
Books
DEFRA Code of Practice or similar guidelines
Other

58.1.a - Source of information

Riding instructor
Another veterinarian
Farrier
Yard owner
Equine charity
Equine physiotherapist
Animal behaviourist
Tack shop worker
Horse owner
Course I have followed / am following
Equine magazine
Internet forums
Online searches
Scientific journals

Books
DEFRA Code of Practice or similar guidelines
Other

58.2.a - Source of information

Riding instructor
Another veterinarian
Farrier
Yard owner
Equine charity
Equine physiotherapist
Animal behaviourist
Tack shop worker
Horse owner
Course I have followed / am following
Equine magazine
Internet forums
Online searches
Scientific journals
Books
DEFRA Code of Practice or similar guidelines
Other

58.3.a - Source of information

Riding instructor
Another veterinarian
Farrier
Yard owner
Equine charity
Equine physiotherapist
Animal behaviourist
Tack shop worker
Horse owner
Course I have followed / am following
Equine magazine
Internet forums
Online searches
Scientific journals
Books
DEFRA Code of Practice or similar guidelines
Other

58.4.a - Source of information

Riding instructor
Another veterinarian
Farrier
Yard owner
Equine charity
Equine physiotherapist
Animal behaviourist
Tack shop worker
Horse owner
Course I have followed / am following
Equine magazine
Internet forums
Online searches
Scientific journals
Books
DEFRA Code of Practice or similar guidelines
Other

59.1.a - Source of information

Riding instructor
Another veterinarian
Farrier
Yard owner
Equine charity
Equine physiotherapist
Animal behaviourist
Tack shop worker
Horse owner
Course I have followed / am following
Equine magazine
Internet forums
Online searches
Scientific journals
Books
DEFRA Code of Practice or similar guidelines
Other

59.2.a - Source of information

Riding instructor
Another veterinarian
Farrier
Yard owner

- Equine charity
- Equine physiotherapist
- Animal behaviourist
- Tack shop worker
- Horse owner
- Course I have followed / am following
- Equine magazine
- Internet forums
- Online searches
- Scientific journals
- Books
- DEFRA Code of Practice or similar guidelines
- Other

59.3.a - Source of information

- Riding instructor
- Another veterinarian
- Farrier
- Yard owner
- Equine charity
- Equine physiotherapist
- Animal behaviourist
- Tack shop worker
- Horse owner
- Course I have followed / am following
- Equine magazine
- Internet forums
- Online searches
- Scientific journals
- Books
- DEFRA Code of Practice or similar guidelines
- Other

59.4.a - Source of information

- Riding instructor
- Another veterinarian
- Farrier
- Yard owner
- Equine charity
- Equine physiotherapist
- Animal behaviourist
- Tack shop worker
- Horse owner

Course I have followed / am following
Equine magazine
Internet forums
Online searches
Scientific journals
Books
DEFRA Code of Practice or similar guidelines
Other

60.1.a - Source of information

Riding instructor
Another veterinarian
Farrier
Yard owner
Equine charity
Equine physiotherapist
Animal behaviourist
Tack shop worker
Horse owner
Course I have followed / am following
Equine magazine
Internet forums
Online searches
Scientific journals
Books
DEFRA Code of Practice or similar guidelines
Other

60.2.a - Source of information

Riding instructor
Another veterinarian
Farrier
Yard owner
Equine charity
Equine physiotherapist
Animal behaviourist
Tack shop worker
Horse owner
Course I have followed / am following
Equine magazine
Internet forums
Online searches
Scientific journals

Books
DEFRA Code of Practice or similar guidelines
Other

60.3.a - Source of information

Riding instructor
Another veterinarian
Farrier
Yard owner
Equine charity
Equine physiotherapist
Animal behaviourist
Tack shop worker
Horse owner
Course I have followed / am following
Equine magazine
Internet forums
Online searches
Scientific journals
Books
DEFRA Code of Practice or similar guidelines
Other

60.4.a - Source of information

Riding instructor
Another veterinarian
Farrier
Yard owner
Equine charity
Equine physiotherapist
Animal behaviourist
Tack shop worker
Horse owner
Course I have followed / am following
Equine magazine
Internet forums
Online searches
Scientific journals
Books
DEFRA Code of Practice or similar guidelines
Other

61.1.a - Source of information

Riding instructor
Another veterinarian
Farrier
Yard owner
Equine charity
Equine physiotherapist
Animal behaviourist
Tack shop worker
Horse owner
Course I have followed / am following
Equine magazine
Internet forums
Online searches
Scientific journals
Books
DEFRA Code of Practice or similar guidelines
Other

61.2.a - Source of information

Riding instructor
Another veterinarian
Farrier
Yard owner
Equine charity
Equine physiotherapist
Animal behaviourist
Tack shop worker
Horse owner
Course I have followed / am following
Equine magazine
Internet forums
Online searches
Scientific journals
Books
DEFRA Code of Practice or similar guidelines
Other

61.3.a - Source of information

Riding instructor
Another veterinarian
Farrier
Yard owner

- Equine charity
- Equine physiotherapist
- Animal behaviourist
- Tack shop worker
- Horse owner
- Course I have followed / am following
- Equine magazine
- Internet forums
- Online searches
- Scientific journals
- Books
- DEFRA Code of Practice or similar guidelines
- Other

61.4.a - Source of information

- Riding instructor
- Another veterinarian
- Farrier
- Yard owner
- Equine charity
- Equine physiotherapist
- Animal behaviourist
- Tack shop worker
- Horse owner
- Course I have followed / am following
- Equine magazine
- Internet forums
- Online searches
- Scientific journals
- Books
- DEFRA Code of Practice or similar guidelines
- Other

62.1.a - Source of information

- Riding instructor
- Another veterinarian
- Farrier
- Yard owner
- Equine charity
- Equine physiotherapist
- Animal behaviourist
- Tack shop worker
- Horse owner

- Course I have followed / am following
- Equine magazine
- Internet forums
- Online searches
- Scientific journals
- Books
- DEFRA Code of Practice or similar guidelines
- Other

62.2.a - Source of information

- Riding instructor
- Another veterinarian
- Farrier
- Yard owner
- Equine charity
- Equine physiotherapist
- Animal behaviourist
- Tack shop worker
- Horse owner
- Course I have followed / am following
- Equine magazine
- Internet forums
- Online searches
- Scientific journals
- Books
- DEFRA Code of Practice or similar guidelines
- Other

62.3.a - Source of information

- Riding instructor
- Another veterinarian
- Farrier
- Yard owner
- Equine charity
- Equine physiotherapist
- Animal behaviourist
- Tack shop worker
- Horse owner
- Course I have followed / am following
- Equine magazine
- Internet forums
- Online searches
- Scientific journals

Books
DEFRA Code of Practice or similar guidelines
Other

62.4.a - Source of information

Riding instructor
Another veterinarian
Farrier
Yard owner
Equine charity
Equine physiotherapist
Animal behaviourist
Tack shop worker
Horse owner
Course I have followed / am following
Equine magazine
Internet forums
Online searches
Scientific journals
Books
DEFRA Code of Practice or similar guidelines
Other

67.1.a - Source of information

Riding instructor
Veterinarian
Farrier
Yard owner
Equine charity
Equine physiotherapist
Animal behaviourist
Tack shop worker
Another horse owner
Course I have followed / am following
Equine magazine
Internet forums
Online searches
Scientific journals
Books
DEFRA Code of Practice or similar guidelines
Other

67.2.a - Source of information

Riding instructor
Veterinarian
Farrier
Yard owner
Equine charity
Equine physiotherapist
Animal behaviourist
Tack shop worker
Another horse owner
Course I have followed / am following
Equine magazine
Internet forums
Online searches
Scientific journals
Books
DEFRA Code of Practice or similar guidelines
Other

67.3.a - Source of information

Riding instructor
Veterinarian
Farrier
Yard owner
Equine charity
Equine physiotherapist
Animal behaviourist
Tack shop worker
Another horse owner
Course I have followed / am following
Equine magazine
Internet forums
Online searches
Scientific journals
Books
DEFRA Code of Practice or similar guidelines
Other

67.4.a - Source of information

Riding instructor
Veterinarian
Farrier
Yard owner

Equine charity
Equine physiotherapist
Animal behaviourist
Tack shop worker
Another horse owner
Course I have followed / am following
Equine magazine
Internet forums
Online searches
Scientific journals
Books
DEFRA Code of Practice or similar guidelines
Other

68.1.a - Source of information

Riding instructor
Veterinarian
Farrier
Yard owner
Equine charity
Equine physiotherapist
Animal behaviourist
Tack shop worker
Another horse owner
Course I have followed / am following
Equine magazine
Internet forums
Online searches
Scientific journals
Books
DEFRA Code of Practice or similar guidelines
Other

68.2.a - Source of information

Riding instructor
Veterinarian
Farrier
Yard owner
Equine charity
Equine physiotherapist
Animal behaviourist
Tack shop worker
Another horse owner

Course I have followed / am following
Equine magazine
Internet forums
Online searches
Scientific journals
Books
DEFRA Code of Practice or similar guidelines
Other

68.3.a - Source of information

Riding instructor
Veterinarian
Farrier
Yard owner
Equine charity
Equine physiotherapist
Animal behaviourist
Tack shop worker
Another horse owner
Course I have followed / am following
Equine magazine
Internet forums
Online searches
Scientific journals
Books
DEFRA Code of Practice or similar guidelines
Other

68.4.a - Source of information

Riding instructor
Veterinarian
Farrier
Yard owner
Equine charity
Equine physiotherapist
Animal behaviourist
Tack shop worker
Another horse owner
Course I have followed / am following
Equine magazine
Internet forums
Online searches
Scientific journals

Books
DEFRA Code of Practice or similar guidelines
Other

69.1.a - Source of information

Riding instructor
Veterinarian
Farrier
Yard owner
Equine charity
Equine physiotherapist
Animal behaviourist
Tack shop worker
Another horse owner
Course I have followed / am following
Equine magazine
Internet forums
Online searches
Scientific journals
Books
DEFRA Code of Practice or similar guidelines
Other

69.2.a - Source of information

Riding instructor
Veterinarian
Farrier
Yard owner
Equine charity
Equine physiotherapist
Animal behaviourist
Tack shop worker
Another horse owner
Course I have followed / am following
Equine magazine
Internet forums
Online searches
Scientific journals
Books
DEFRA Code of Practice or similar guidelines
Other

69.3.a - Source of information

- Riding instructor
- Veterinarian
- Farrier
- Yard owner
- Equine charity
- Equine physiotherapist
- Animal behaviourist
- Tack shop worker
- Another horse owner
- Course I have followed / am following
- Equine magazine
- Internet forums
- Online searches
- Scientific journals
- Books
- DEFRA Code of Practice or similar guidelines
- Other

69.4.a - Source of information

- Riding instructor
- Veterinarian
- Farrier
- Yard owner
- Equine charity
- Equine physiotherapist
- Animal behaviourist
- Tack shop worker
- Another horse owner
- Course I have followed / am following
- Equine magazine
- Internet forums
- Online searches
- Scientific journals
- Books
- DEFRA Code of Practice or similar guidelines
- Other

70.1.a - Source of information

- Riding instructor
- Veterinarian
- Farrier
- Yard owner

- Equine charity
- Equine physiotherapist
- Animal behaviourist
- Tack shop worker
- Another horse owner
- Course I have followed / am following
- Equine magazine
- Internet forums
- Online searches
- Scientific journals
- Books
- DEFRA Code of Practice or similar guidelines
- Other

70.2.a - Source of information

- Riding instructor
- Veterinarian
- Farrier
- Yard owner
- Equine charity
- Equine physiotherapist
- Animal behaviourist
- Tack shop worker
- Another horse owner
- Course I have followed / am following
- Equine magazine
- Internet forums
- Online searches
- Scientific journals
- Books
- DEFRA Code of Practice or similar guidelines
- Other

70.3.a - Source of information

- Riding instructor
- Veterinarian
- Farrier
- Yard owner
- Equine charity
- Equine physiotherapist
- Animal behaviourist
- Tack shop worker
- Another horse owner

Course I have followed / am following
Equine magazine
Internet forums
Online searches
Scientific journals
Books
DEFRA Code of Practice or similar guidelines
Other

70.4.a - Source of information

Riding instructor
Veterinarian
Farrier
Yard owner
Equine charity
Equine physiotherapist
Animal behaviourist
Tack shop worker
Another horse owner
Course I have followed / am following
Equine magazine
Internet forums
Online searches
Scientific journals
Books
DEFRA Code of Practice or similar guidelines
Other

71.1.a - Source of information

Riding instructor
Veterinarian
Farrier
Yard owner
Equine charity
Equine physiotherapist
Animal behaviourist
Tack shop worker
Another horse owner
Course I have followed / am following
Equine magazine
Internet forums
Online searches
Scientific journals

Books
DEFRA Code of Practice or similar guidelines
Other

71.2.a - Source of information

Riding instructor
Veterinarian
Farrier
Yard owner
Equine charity
Equine physiotherapist
Animal behaviourist
Tack shop worker
Another horse owner
Course I have followed / am following
Equine magazine
Internet forums
Online searches
Scientific journals
Books
DEFRA Code of Practice or similar guidelines
Other

71.3.a - Source of information

Riding instructor
Veterinarian
Farrier
Yard owner
Equine charity
Equine physiotherapist
Animal behaviourist
Tack shop worker
Another horse owner
Course I have followed / am following
Equine magazine
Internet forums
Online searches
Scientific journals
Books
DEFRA Code of Practice or similar guidelines
Other

71.4.a - Source of information

- Riding instructor
- Veterinarian
- Farrier
- Yard owner
- Equine charity
- Equine physiotherapist
- Animal behaviourist
- Tack shop worker
- Another horse owner
- Course I have followed / am following
- Equine magazine
- Internet forums
- Online searches
- Scientific journals
- Books
- DEFRA Code of Practice or similar guidelines
- Other

72.1.a - Source of information

- Riding instructor
- Veterinarian
- Farrier
- Yard owner
- Equine charity
- Equine physiotherapist
- Animal behaviourist
- Tack shop worker
- Another horse owner
- Course I have followed / am following
- Equine magazine
- Internet forums
- Online searches
- Scientific journals
- Books
- DEFRA Code of Practice or similar guidelines
- Other

72.2.a - Source of information

- Riding instructor
- Veterinarian
- Farrier
- Yard owner

Equine charity
Equine physiotherapist
Animal behaviourist
Tack shop worker
Another horse owner
Course I have followed / am following
Equine magazine
Internet forums
Online searches
Scientific journals
Books
DEFRA Code of Practice or similar guidelines
Other

72.3.a - Source of information

Riding instructor
Veterinarian
Farrier
Yard owner
Equine charity
Equine physiotherapist
Animal behaviourist
Tack shop worker
Another horse owner
Course I have followed / am following
Equine magazine
Internet forums
Online searches
Scientific journals
Books
DEFRA Code of Practice or similar guidelines
Other

72.4.a - Source of information

Riding instructor
Veterinarian
Farrier
Yard owner
Equine charity
Equine physiotherapist
Animal behaviourist
Tack shop worker
Another horse owner

Course I have followed / am following
Equine magazine
Internet forums
Online searches
Scientific journals
Books
DEFRA Code of Practice or similar guidelines
Other
